# Engineering Light‐Element Modified LaFe_11.6_Si_1.4_ Compounds Enables Tunable Giant Magnetocaloric Effect

**DOI:** 10.1002/advs.202416288

**Published:** 2025-05-19

**Authors:** Fengqi Zhang, Ziying Wu, Xiaofang Zhang, Xiang Chi, Zhenduo Wu, Jianrong Gao, Huaican Chen, Wen Yin, Ulrich Lienert, Ann‐Christin Dippel, Martin v. Zimmermann, Niels van Dijk, Ekkes Brück, Yang Ren

**Affiliations:** ^1^ JC STEM Lab of Energy and Materials Physics Department of Physics City University of Hong Kong Kowloon Hong Kong SAR 999077 China; ^2^ Fundamental Aspects of Materials and Energy (FAME) Faculty of Applied Sciences Delft University of Technology Mekelweg 15 2629JB Delft The Netherlands; ^3^ Songshan Lake Materials Laboratory 523775 Dongguan China; ^4^ City University of Hong Kong (Dongguan) 523808 Dongguan China; ^5^ Key Laboratory of Electromagnetic Processing of Materials (Ministry of Education) Northeastern University Shenyang 110819 China; ^6^ Spallation Neutron Source Science Center Dalang Dongguan 523803 China; ^7^ Institute of High Energy Physics Chinese Academy of Sciences Beijing 100049 China; ^8^ Deutsches Elektronen‐Synchrotron DESY Notkestraße 85 22607 Hamburg Germany; ^9^ Center for Neutron Scattering City University of Hong Kong Kowloon Hong Kong SAR 999077 China

**Keywords:** La(Fe,Si)_13_, light element doping, magnetocaloric energy conversions, magnetocaloric materials, synchrotron X‐ray and neutron diffractions

## Abstract

Magnetocaloric refrigeration is one of the most promising next‐generation solid‐state caloric techniques to revolutionize the traditional air‐compression technique. The La(Fe,Si)_13_‐based materials are recognized as candidates with potential for practical applications. However, flexible strategies to improve the Curie temperature (*T_C_*) and further achieve the tunable giant magnetocaloric effect (GMCE) still need to be developed. Here, the systematic experimental investigation on a series of light elements (C, F, S) modified LaFe_11.6_Si_1.4_ compounds are presented. It is found that all modified samples exhibit a higher *T_C_*, with a negligible impact on the thermal hysteresis. The GMCE performance in C‐ and S‐modified samples is significantly degraded, but the maximum magnetic entropy change |*Δ s_m_* | for the optimally doped F sample can be well maintained at 19.2 J kg^−1^ K^−1^ for a field change of 2 T. The preferential site occupancy of dopants is determined, and the microstructural observation and metastable atomic changes have also been analyzed. It is concluded that interstitial doping is more efficient to shift *T_C_*. The first‐order transition can however not be maintained upon doping due to changes in the hybridization. These findings highlight the importance of the interplay between the lattice pressure effect and the covalent hybridization for this material family.

## Graphical abstract

Herein, a tunable magnetocaloric effect is achieved on a series of light elements (C, F, S) modified LaFe_11.6_Si_1.4_ compounds. It is found that interstitial doping is more efficient in shifting *T_C_* compared with mixed (substitutional/interstitial) doping, but the first‐order transition cannot be well kept because of different *p–d* hybridization.

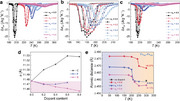

## 1 Introduction

The widely equipped vapor‐compression technique for heating‐ventilation air‐conditioning (HVAC) accelerates the urgency of reducing the overall carbon footprint, where emerging solid‐state caloric technologies provide promising resolution strategies for these issues.^[^ ^1^, ^2^ ^]^ Within these techniques, the magnetocaloric refrigeration (McR) technique, which applies magnetocaloric effect materials (MCEMs), has developed rapidly in recent years.^[^ ^3^, ^4^ ^]^ This technique requires no greenhouse gas as a working medium and has a higher energy utilization efficiency.^[^ ^5^, ^6^ ^]^ The MCEMs with a first‐order magnetic transition (FOMT) create a noticeable isothermal entropy change and an adiabatic temperature change (Δ*T_ad_*), which makes them suitable for McR.^[^ ^7^ ^]^ Distinguishing by the so‐called magneto‐elastic coupling and magneto‐structural coupling mechanism, which result in a giant magnetocaloric effect (GMCE) within MCEMs, several representative promising candidate materials have been found including Gd_5_(Si_2_Ge_2_),^[^ ^8^ ^]^ La(Fe,Si)_13_ based materials,^[^ ^9^, ^10^, ^11^, ^12^, ^13^, ^14^ ^]^ (Mn,Fe)_2_(P,*X*) based compounds (*X* = As, Ge, Si),^[^ ^15^, ^16^ ^]^ NiMn‐*X* based magnetic Heusler alloys (*X* = Al, Ga, In, Sn, Sb, (Co)Ti),^[^ ^17^, ^18^ ^]^ Mn*M*‐*X* (*M* = Co or Ni, *X* = Si or Ge) ferromagnets,^[^ ^19^, ^20^ ^]^ and FeRh.^[^ ^21^ ^]^ Within these MCEMs, the NaZn_13_‐type La(Fe,Si)_13_‐based compounds with a cubic crystal structure present an iso‐structural FOMT and show a strong magnetoelastic coupling among the magnetic, structural and electronic degrees of freedom.^[^ ^22^ ^]^ Considerable attention has been paid to this system due to advantages such as: excellent GMCE performance, no toxic elements, low material cost, low criticality, a tunable Curie temperature (*T_C_*), and other fruitful physical function properties.^[^ ^23^, ^24^, ^25^ ^]^

To further optimize its GMCE properties, different optimization strategies have been applied for La(Fe,Si)_13_‐based materials like tuning the metallic and nonmetallic ratios,^[^ ^26^, ^27^, ^28^, ^29^ ^]^ substitutional/interstitial doping,^[^ ^30^, ^31^, ^32^, ^33^, ^34^, ^35^, ^36^, ^37^, ^38^, ^39^, ^40^, ^41^, ^42^ ^]^ advanced manufacturing,^[^ ^43^, ^44^, ^45^, ^46^, ^47^ ^]^ composite engineering,^[^ ^48^, ^49^, ^50^ ^]^ etc. For example, the binder‐free laser powder bed fusion (LPBF) has been employed to fabricate 3D channel or pillar structures,^[^ ^46^, ^47^ ^]^ where a magnetic entropy change (|Δ*s_m_* |) for printed materials of 17 J kg^−1^ K^−1^ can be achieved for a field change of *Δ µ*_0_ *H* = 1 T.^[^ ^47^ ^]^ Liu et al. applied hot‐rolling to manipulate the microstructure of LaFe_11.6_Si_1.4_/Fe composites and found that |Δ*s_m_* | was maintained at 17 J kg^−1^ K^−1^ for *Δ μ*_0_ *H* = 2 T, with an enhanced mechanical strength (bending strength of 176 MPa).^[^ ^50^ ^]^ Moreover, through co‐doping of Ce and H atoms, the thermal hysteresis in (La_1‐_ *_x_* Ce*_x_*)_2_Fe_11_Si_2_H*_y_* materials is monotonously reduced and a large Δ*T_ad_* of 2.03 K in 1.3 T can be obtained after 10^5^ magnetic cycles.^[^ ^36^ ^]^ However, to positively adjust *T_C_* towards room temperature (RT), almost all produced materials heavily rely on the absorption of H under pressure. Unfortunately, one unavoidable problem for the hydrides is their chemical instability above 330 K, which is detrimental to practical applications.^[^ ^51^, ^52^ ^]^ Furthermore, many studies have been devoted to the increase in *T_C_*, e.g. substitutional doping with Ni^[^ ^33^ ^]^ and Co.^[^ ^53^, ^54^ ^]^ Note that almost all metallic dopants cause a decrease in *T_C_*.^[^ ^34^, ^35^, ^55^, ^56^, ^57^ ^]^ As observed for the (Mn,Fe)_2_(P,Si) materials,^[^ ^58^, ^59^ ^]^ the interstitial doping of *p*‐block elements like C can be an effective way to raise the phase transition temperature near RT.^[^ ^30^, ^60^, ^61^ ^]^ Nevertheless, systematic experimental investigations on the influence of light elements such as C, F, and S for the structural and magnetic property changes in La(Fe,Si)_13_‐based compounds are still lacking, which necessitates the current study. Here we report that different series of dopants (C, F, S) modified LaFe_11.6_Si_1.4_ compounds have been produced and their thermodynamic properties, magnetic properties including GMCE, microstructural observations, and atomic‐scale changes have been investigated. In comparison with the undoped sample, it is found that with increasing dopant contents all modified samples exhibit a higher *T_C_*, with a negligible impact on the thermal hysteresis (Δ*T_hys_*). Meanwhile, for higher doping contents the GMCE performance in C‐ and S‐modified samples is significantly degraded towards a second‐order magnetic transition (SOMT). However, the maximum |*Δ s_m_* | for Δ *μ*_0_ *H* = 2 T in optimally doped F sample is maintained at 19.2 J kg^−1^ K^−1^. Combining Electron Probe Microanalysis (EPMA), high‐resolution transmission electron microscopy (HR‐TEM), temperature‐dependent synchrotron X‐ray diffraction (XRD), and neutron diffraction (ND), the preferential site occupancy of these light element dopants has been determined. The corresponding microstructural observations and metastable atomic changes among different atom pairs across the transition have been studied. It is concluded that interstitial doping is more efficient for the shift in *T_C_* compared with mixed (substitutional/interstitial) doping, but it is not beneficial to maintain the FOMT due to changes in the hybridization. The results further highlight the importance of the interplay between the anomalous chemical pressure effect on the lattice and the covalent hybridization for the tunable itinerant‐electron metamagnetic transition in the La(Fe,Si)_13_‐based compounds. It further deepens our understanding and reinforces the potential of this material family for solid‐state caloric applications.

## 2 Results and Discussion

**Figure 1:**
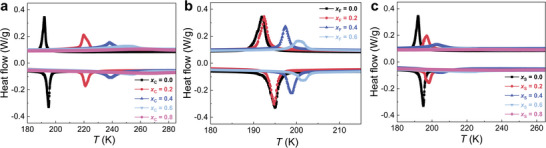
Heat flow derived from DSC experiments upon warming and cooling processes at a rate of 10 K/min. a) LaFe_11.6_Si_1.4_C*_x_*_C_ (*x*_C_ = 0.0, 0.2, 0.4, 0.6, 0.8), b) LaFe_11.6_Si_1.4_F*_x_*_F_ (*x*_F_ = 0.0, 0.2, 0.4, 0.6), c) LaFe_11.6_Si_1.4_S*_x_*_S_ (*x*_S_ = 0.0, 0.2, 0.4, 0.6, 0.8).

The heat flow as a function of temperature measured by zero‐field DSC for the modified LaFe_11.6_Si_1.4_ samples is presented in **Figure** **1**. It is observed that for LaFe_11.6_Si_1.4_F*_x_*_F_ (*x*_F_ = 0.0, 0.2, 0.4, 0.6) samples the FOMT nature is maintained after modification. For comparison, as shown in Figure 1a,c, however, this characteristic for the LaFe_11.6_Si_1.4_C*_x_*_C_ (*x*_C_ = 0.0, 0.2, 0.4, 0.6, 0.8) and LaFe_11.6_Si_1.4_S*_x_*_S_ (*x*_S_ = 0.0, 0.2, 0.4, 0.6, 0.8) samples has been reduced because of the continuous disappearance of the endothermic and exothermic peaks, especially for higher dopant contents. Interestingly, for all light element modifications, the *T_C_* continuously increases with increasing the dopant concentration. For instance, for F doping *T_C_* shifts up from 195.0 (*x*_F_ = 0.0) to 201.6 K (*x*_F_ = 0.6), with an increment of 1.6 K/at.% dopant. The increment for C, F, and S modified LaFe_11.6_Si_1.4_ compounds are ≈13.7, 1.6, and 9.0 K/*at*.% dopant. It is noted that C presents the most significant enhancement in *T_C_*, which is in good agreement with previous studies (≈ 12.5 K/at. % C).^[^ ^60^ ^]^ After modification, the *Δ T_hys_* values of the doped materials are comparable to the parent compound. A very low *Δ T_hys_* will be beneficial for the reversibility of magnetocaloric cycles as *Δ T_hys_* can significantly decrease the energy efficiency of cooling devices.^[^ ^62^ ^]^ In addition, the multi‐cycle DSC experiments for *x*_F_ = 0.4 sample in Figure S1 (Supporting Information) indicate that the sample presents very good stability and reversibility at high temperatures (373 K). From the DSC results, the characteristic temperatures such as *T_C_* upon cooling and heating (*T_C_* ^cooling^ and *T_C_* ^heating^) and *Δ T_hys_* for all mentioned samples are extracted and summarized in **Table** **1**.

**Table 1:** Summary of *T_C_* upon cooling (*T_C_^cooling^* DSC and *T_C_^cooling^* SQUID), the *T_C_* upon heating (*T_C_^heating^* DSC and *T_C_^heating^* SQUID), the *Δ T_hys_* (*Δ T_hys_^DSC^* and *Δ T_hys_^SQUID^*), the absolute magnetic entropy change upon heating |*Δ s_m_^heating^|* in different magnetic field changes Δ*µ*_0_ *H* = 1(2)T for different samples.

Sample	*Tc* ^cooling^ DSC [K]	*Tc* ^cooling^ SQUID [K]	*Tc* ^heating^ DSC [K]	*Tc* ^heating^ SQUID [K]	Δ*T_hys_* ^DSC^ [K]	Δ*T_hys_* ^SQUID^ [K]	|Δ*s_m_^heating^* | [J kg^−1^ K^−1^]
No dopant	192.0	196.1	195.0	196.6	3.0	0.5	22.4(25.6)
*x*_C_ = 0.2	219.8	227.4	221.0	227.4	1.2	0.0	13.7(17.2)
*x*_C_ = 0.4	238.1	242.2	239.0	242.6	0.9	0.4	7.9(11.6)
*x*_C_ = 0.6	253.4	254.7	254.2	254.7	0.8	0.0	3.0(5.3)
*x*_C_ = 0.8	269.1	272.7	269.1	272.7	0.0	0.0	1.4(2.6)
*x*_F_ = 0.2	192.5	195.8	194.8	196.6	2.3	0.8	21.3(23.4)
*x*_F_ = 0.4	197.4	199.3	198.8	200.0	1.4	0.7	16.7(19.2)
*x*_F_ = 0.6	200.7	202.2	201.6	202.8	0.9	0.6	10.1(13.3)
*x*_S_ = 0.2	196.9	198.2	198.3	198.8	1.4	0.6	13.2(15.9)
*x*_S_ = 0.4	202.3	208.5	204.8	208.5	2.5	0.0	3.1(5.7)
*x*_S_ = 0.6	224.0	224.4	224.0	224.4	0.0	0.0	1.3(2.5)
*x*_S_ = 0.8	243.9	244.8	243.9	244.8	0.0	0.0	1.0(1.9)

**Figure 2:**
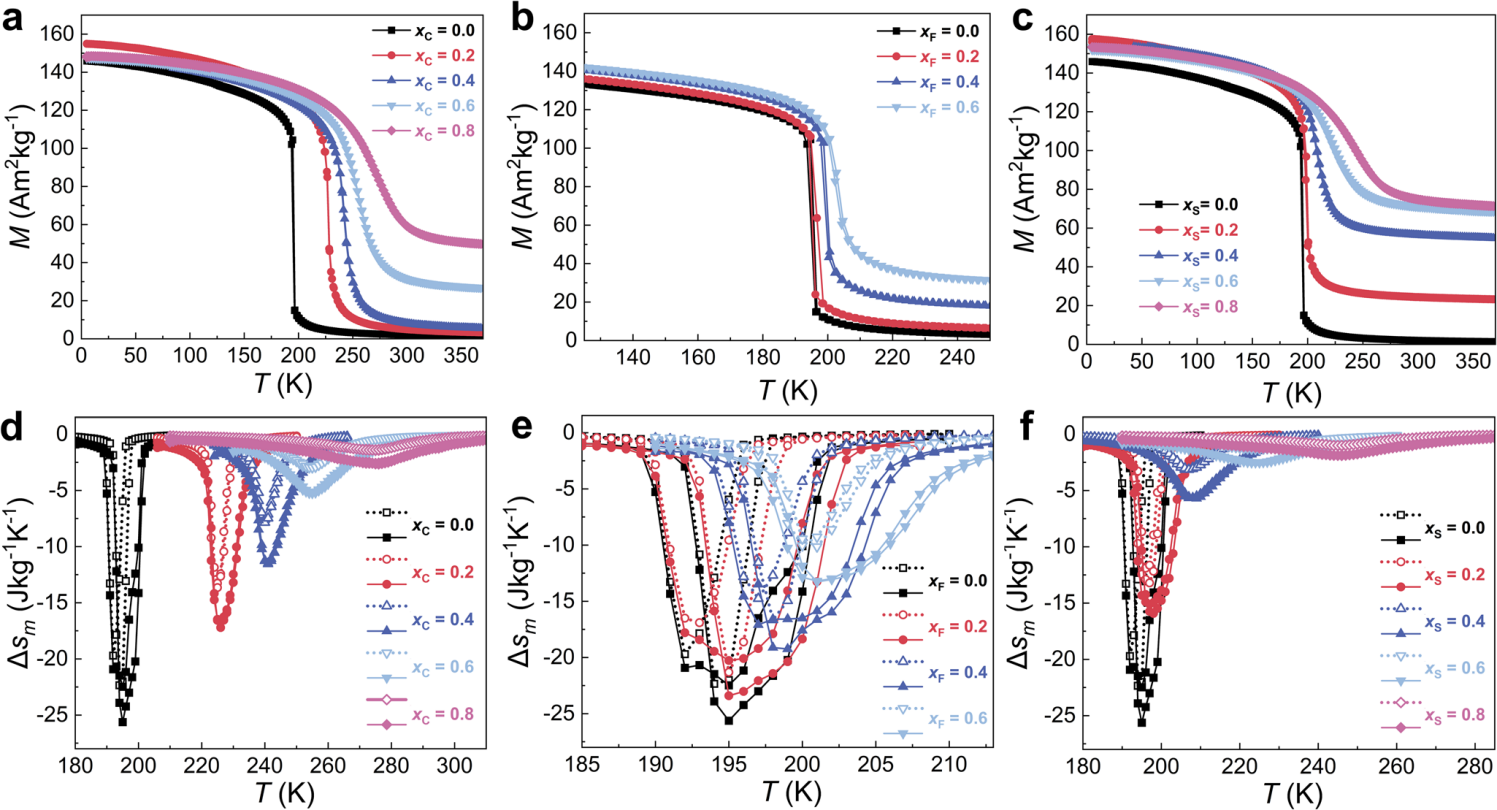
Iso‐field *M‐T* curves for different doped LaFe_11.6_Si_1.4_ samples in 1 T. a) LaFe_11.6_Si_1.4_C*_x_*_C_ (*x*_C_ = 0.0, 0.2, 0.4, 0.6, 0.8), b) LaFe_11.6_Si_1.4_F*_x_*_F_ (*x*_F_ = 0.0, 0.2, 0.4, 0.6), c) LaFe_11.6_Si_1.4_S*_x_*_S_ (*x*_S_ = 0.0, 0.2, 0.4, 0.6, 0.8). *Δ s*_m_ of different doped samples as a function of temperature for both heating and cooling processes, determined for *Δ µ*_0_ *H* ranging from 1 (open symbol) to 2 T (solid symbol). d) LaFe_11.6_Si_1.4_C*_x_*_C_ (*x*_C_ = 0.0, 0.2, 0.4, 0.6, 0.8), e) LaFe_11.6_Si_1.4_F*_x_*_F_ (*x*_F_ = 0.0, 0.2, 0.4, 0.6), (f) LaFe_11.6_Si_1.4_S*_x_*_S_ (*x*_S_ = 0.0, 0.2, 0.4, 0.6, 0.8).

The iso‐field *M–T* curves for these samples are measured in an applied magnetic field of 1 T, as shown in **Figure** **2**a–c. A FOMT from the low‐temperature FM to high‐temperature PM state is observed, with phase transitions located between 180 and 300 K. *T_C_* for all modified samples show positive shifts, which indicates an enhancement of magnetic exchange interactions, considering the proportionality between *T_C_* and exchange interactions (within the mean field approximation (MFA)).^[^ ^63^ ^]^ For instance, in comparison with the parent compound with *T_C_^heating^* = 196.6 K, the value for *T_C_^heating^* for different dopants can reach 272.7 K (*x*_C_ = 0.8), 202.8 K (*x*_F_ = 0.6) and 244.8 K (*x*_S_ = 0.8), respectively. Taking other MCEMs with strong FOMT properties like Fe_2_P‐type, Mn*MX*, FeRh, and Eu_2_In as examples,^[^ ^20^, ^59^, ^64^, ^65^ ^]^ the magnetoelastic coupling is governed by the magnetic exchange interactions, as well as the hybridization among various atoms. In addition, compared with the well‐known H insertion for La(Fe,Si)_13_‐based compounds,^[^ ^66^ ^]^ the differences in *T_C_* changes upon C, F, and S modification could be ascribed to different interstitial or substitutional doping mechanisms (will be discussed in the following sections), which has also been observed in (Mn,Fe)_2_(P,Si) based MCEMs.^[^ ^58^, ^59^ ^]^ It is suggested that the modification of electronic structure upon different light element doping (e.g., H, B, C, N, F, and S) for La(Fe,Si)_13_‐based materials is crucial, as well as the normal chemical pressure effects.^[^ ^61^ ^]^ Additionally, the enhanced magnetization especially for higher dopant contents in the PM state could be attributed to the concentration fluctuation in the *α*‐Fe phase.^[^ ^33^, ^67^ ^]^ Moreover, Figure 2 shows that with increasing doping content of C and S the FOMT is shifting to SOMT due to the non‐hysteretic characteristics, while for F the FOMT could become a weak FOMT or a critical point between FOMT and SOMT because of the low *Δ T_hys_* (even <1 K). It is worth mentioning that the lower *Δ T_hys_* for the SQUID (2 K per min) in comparison to the DSC (10 K per min) measurements could be ascribed to the slower heating–cooling rate because the first‐order transitions are driven by nucleation and growth, and the time‐dependent transitions need a certain response time.^[^ ^68^ ^]^ The characteristic temperatures of *T_C_^cooling^* and *T_C_^heating^*, and *Δ T_hys_* for all samples extracted from magnetic measurement are combined into Table 1.

To further evaluate the GMCE performance of the above samples, Figure 2d–f presents the calculated *Δ s_m_* values for cooling and heating processes up to a practical field change of 0–2 T (limitation from Nd‐Fe‐B permanent magnet source) as a function of temperature, in the vicinity of the phase transition. The *Δ s_m_* with different field changes Δ *μ*_0_ *H* can be calculated using the Maxwell relation by:^[^ ^69^ ^]^

(1) $$▵s_{m}\left(T,H\right)=\int_{0}^{H}{\left(\frac{\partial M}{\partial T}\right)}_{H}d\mu_{0}H$$

It is illustrated that all samples show a conventional GMCE upon applying a magnetic field. It is noted that the parent compound without dopants shows the largest |Δ s_m_| peak (for heating) at 22.4(25.6) J kg^−1^ K^−1^ for Δ *μ*_0_ *H* = 1(2) T. However, the maximum |*Δ s_m_* | for the *x*_C_ = 0.4 and *x*_S_ = 0.4 samples shows a significant reduction of around 54.7% (11.6 J kg^−1^ K^−1^) and 77.7% (5.7 J kg^−1^ K^−1^) for Δ *μ*_0_ *H* = 2 T. With increasing *T_C_*, the maximum |Δ s_m_| with Δ *μ*_0_ *H* = 1(2) T only reaches 1.4(2.6) and 1.0(1.9) J kg^−1^ K^−1^ for *x*_C_ = 0.8 and *x*_S_ = 0.8 samples, respectively, and the GMCE for these samples almost vanishes. Note that the reduced |*Δ s_m_|* upon C doping is in good agreement with previous studies.^[^ ^60^, ^70^ ^]^ Meanwhile, the decrease in the maximum |*Δ s_m_* | for the *x*_F_ = 0.4 samples with Δ *μ*_0_ *H* = 2 T is relatively moderate (≈25.0%), as the values are conserved at 19.2 J kg^−1^ K^−1^, respectively. The above‐mentioned findings are unusual as another common light element H in La(Fe,Si)_13_‐based alloys results in an increased *T_C_* and an enhanced |*Δ s_m_* |.^[^ ^31^, ^66^ ^]^ For example, it has been reported previously that H absorption in La(Fe_0.88_Si_0.12_)H*_y_* (*y* = 0.5–1.5) can efficiently enhance *T_C_* from 195 to 323 K, while the excellent GMCE performance resulted in a |*Δ s_m_* | of ≈19–20 J kg^−1^ K^−1^ for Δ *μ*_0_ *H* = 2 T in wide temperature range without obvious degradation.^[^ ^66^ ^]^ Considering the difference in free electrons for C (1s^2^2s^2^2p^2^), F (1s^2^2s^2^2p^5^), and S (3s^2^3p^4^) and elemental electronegativity (*χ*_C _≈ 2.5, *χ*_F_ ≈ 4.0 and *χ*_S_ ≈ 2.5) among these light elements,^[^ ^71^ ^]^ the changes in electronic structures and ferromagnetic exchange coupling are expected to be responsible for the difference in magnetic response for these doped materials.

**Figure 3:**
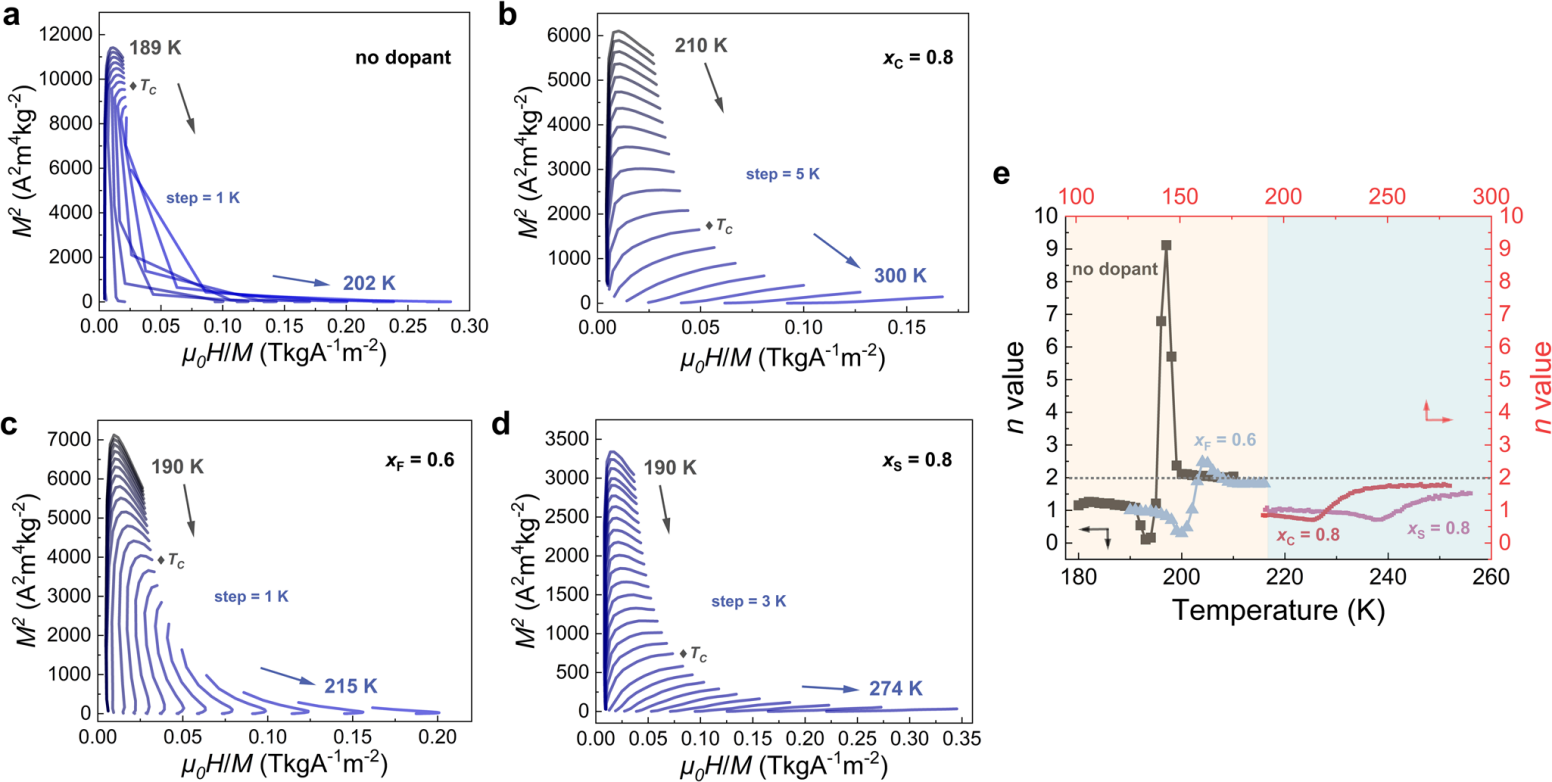
Arrott plots for the selected samples: a) undoped, b) *x*_C_ = 0.8, c) *x*_F_ = 0.6, d) *x*_S_ = 0.8. The magnetic impurities have been considered. The positions of *T_C_* have been marked as ♦. e) Corresponding temperature dependence of the field exponent *n* for the selected samples in an applied field of 1 T. The demagnetizing effect has been considered.

With increasing the doping content, it is important to determine the nature of the phase transition for different modified systems, e.g. FOMT, SOMT, or on the border between them (the so‐called critical point CP). Therefore, as shown in **Figure** **3**a–d, the Arrott plots (*μ_0_H*/*M* versus *M* ^2^) extracted from the iso‐thermal *M–H* curves were constructed. On the basis of the Banerjee criterion,^[^ ^72^ ^]^ it is distinguishable that the undoped, *x*_F_ = 0.6 sample represents clear FOMT characteristics (with an “S‐shaped” curve and a negative slope near *T_C_*), as present in Figure 3a,c. Nevertheless, in Figure 3b,d the *x*_C_ = 0.8 and *x*_S_ = 0.8 samples show SOMT characteristics because of the positive slopes near *T_C_*. Furthermore, the recently proposed field exponent *n* for *Δ s_m_* ^[^ ^73^ ^]^ is applied to further identify the nature of the magnetic transition. The field exponent *n* is defined as:^[^ ^73^, ^74^ ^]^

(2) $$n\left(T,H\right)=\frac{dln\left(|\Delta s_{m}|\right)}{dln\left(H\right)}$$

In Figure 3e, the field exponent *n* as a function of temperature at a field value of 1 T has been shown for the five chosen samples. Note that the demagnetizing effect has been considered for the exponent *n* method,^[^ ^73^, ^75^, ^76^, ^77^ ^]^ which shows influence on the values as shown in Figure S2 (Supporting Information). In the low‐temperature region of Figure 3e (yellow shadow area), it is noticed that for the undoped, *x*_F_ = 0.6 sample the values of the exponent *n* stabilize ≈1 when the temperature is well below *T_C_*, which is the expected field dependence of the magnetization at low temperatures,^[^ ^78^, ^79^ ^]^ while the *n* values abruptly overshoot near *T*_C_ and finally tend to a value ≈ 2 well above *T_C_*. In the high‐temperature range of Figure 3e (blue shadow area), it can be seen that the *n* values for the *x*_C_ = 0.8 and *x*_S_ = 0.8 samples are all <2, and importantly it does not show a peak near *T*_C_ at a value above the value obtained for the PM state at a higher temperature, a characteristic feature for a SOMT.^[^ ^80^, ^81^ ^]^ Therefore, it is concluded that doping with a limited amount of C and S for La(Fe,Si)_13_‐based materials will passivate the FOMT to SOMT, while the F‐doped systems maintain the FOMT with a robust magnetoelastic coupling. The above results are well in line with the results from the Arrott plots. It is worth mentioning that the calculated enthalpy change based on DSC results has been presented in Figure S3 (Supporting Information), and interestingly, samples with SOMT show a non‐zero enthalpy change. Note that for the SOMT a shallow lambda‐like cusp characteristic in the heat‐flow/specific‐heat can be observed in some materials,^[^ ^73^, ^82^, ^83^ ^]^ which can be affected by the properties of the samples like disorder, inhomogeneity, and so on.^[^ ^84^, ^85^ ^]^

**Figure 4:**
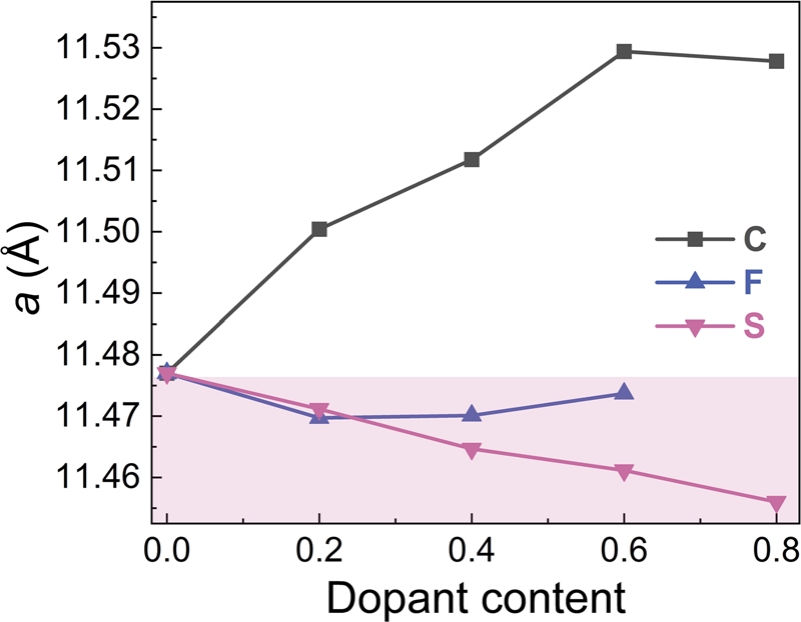
Lattice parameter *a* as a function of the dopant concentration for C, F, and S doping, obtained from XRD data.

Determining which atoms are replaced upon introducing a third element is crucial to further distinguish the subtle structural changes, where the dopants can be addressed through experimental ways or theoretical calculations.^[^ ^86^ ^]^ For the La(Fe,Si)_13_‐based compounds, it is known that the so‐called chemical pressure effect induced by light element interstitial insertion is vital and effective in achieving an increase in *T_C_*.^[^ ^66^, ^87^ ^]^ Considering the differences in the atomic covalent radius for C (0.69 Å), F (0.57 Å) and S (1.05 Å),^[^ ^88^ ^]^ it is reasonable to further investigate the effect of interstitial/substitutional doping and the site occupancy of these atoms in comparison to the host atoms La (2.07 Å), Fe (1.32 Å) and Si (1.11 Å),^[^ ^88^ ^]^ which are crucial in controlling the FOMT.^[^ ^58^, ^59^, ^89^ ^]^ As demonstrated in **Figure** **4**, the lattice parameter *a* obtained from RT XRD measurements shows an obvious difference in behavior between C and the other elements. Compared with F and S doping, it is found that the lattice *a* continuously increases with increasing C content, which confirms that C leads to interstitial doping, resulting in a lattice expansion of the main phase. The result is in good agreement with previous ND experiments, where the site occupancy of the C atoms is determined as the interstitial *24d/48f* positions.^[^ ^90^, ^91^ ^]^ As presented in the shaded area in Figure 4, the experimental data further indicate that: i) the F atoms are likely to enter the main phase by both substitutional and interstitial doping because of the decrease *in a*, which could be ascribed to its small radius. ii) The bigger S atoms are likely to substitute certain host atoms like the non‐metal Si due to their similar covalent radius, resulting in a lattice contraction. The extracted lattice parameter *a* for all the above‐mentioned samples is summarized in Table S1 (Supporting Information).

**Figure 5:**
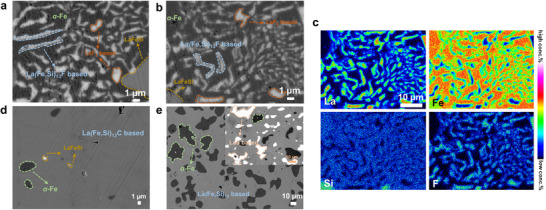
a) Back‐scattered SEM image for the LaFe_11.6_Si_1.4_F_0.4_ sample with different marked impurity phases. b) Back‐scattered SEM images in different regions for the same sample. c) Corresponding FE‐EPMA elemental mapping images for La, Fe, Si, and F elements. Back‐scattered SEM images for the d) LaFe_11.6_Si_1.4_C_0.4_, e) LaFe_11.6_Si_1.4_S_0.4_ samples with a distribution of the different marked impurity phases.

Furthermore, to better understand the phase transition mechanism, the microstructures for the doped samples have been studied. In **Figure** **5**a,b the back‐scattered SEM images for the *x*_F_ = 0.4 sample clearly demonstrate the different impurities including *α*‐Fe, LaFeSi‐ and LaF_2_‐based phases, which are surrounded by the characteristic dendritic shape of the main phase. These excessive impurities are normal for the arc‐melted La(Fe,Si)_13_‐based samples because of the incomplete peritectic reaction.^[^ ^92^ ^]^ Moreover, to observe the existence of doped F within the matrix, as illustrated in Figure 5c (from the selected region in Figure 5b), the different elemental EPMA‐EDS mappings show a homogeneous distribution of La, Fe, Si, and F in the main phase. Similarly, as shown in Figure S4 (Supporting Information), the compositional maps for the selected *x*_C_ = 0.4 sample also present homogeneity of the host elements (La, Fe, Si) as well as C. However, in Figure S5 (Supporting Information) the S atoms cannot be tracked for the *x*_S_ = 0.4 sample. The above results indicate the homogeneous distribution of elements and the introduced light elements (excluding S) have been monitored at a microscopic scale. Meanwhile, EDS maps of the main phase from HR‐TEM measurements are collected at lower dimensions (with a resolution of 100 nm). As demonstrated in Figure S6a–e,f–j (Supporting Information), it is clearly found that C and F, along with the host elements are distinguishable and homogeneously distributed in the main phase. In Figure S6k–o (Supporting Information) S cannot be distinguished, which further proves the exclusiveness of the main phase towards external S atoms. In addition, from Figure 5d a limited amount of *α*‐Fe and LaFeSi‐based phases without other impurities have been found for the *x*_C_ = 0.4 sample. Nevertheless, as shown in Figure 5e, for the corresponding *x*_S_ = 0.4 sample, except for *α*‐Fe phase another LaS‐based impurity (see the inset of Figure 5e) is widely distributed among the main phase (light grey area). This could indicate that the introduced S atoms are mainly located at the LaS‐based impurity. The formed *α*‐Fe and LaS‐based impurities could contribute to the remarkable degradation of the GMCE for the LaFe_11.6_Si_1.4_S_0.4_ sample, as shown in Figure 2f. These results indicate that the introduced light elements are well distributed and highlight the importance of microstructural changes for the GMCE in La(Fe,Si)_13_‐based compounds.

**Figure 6:**
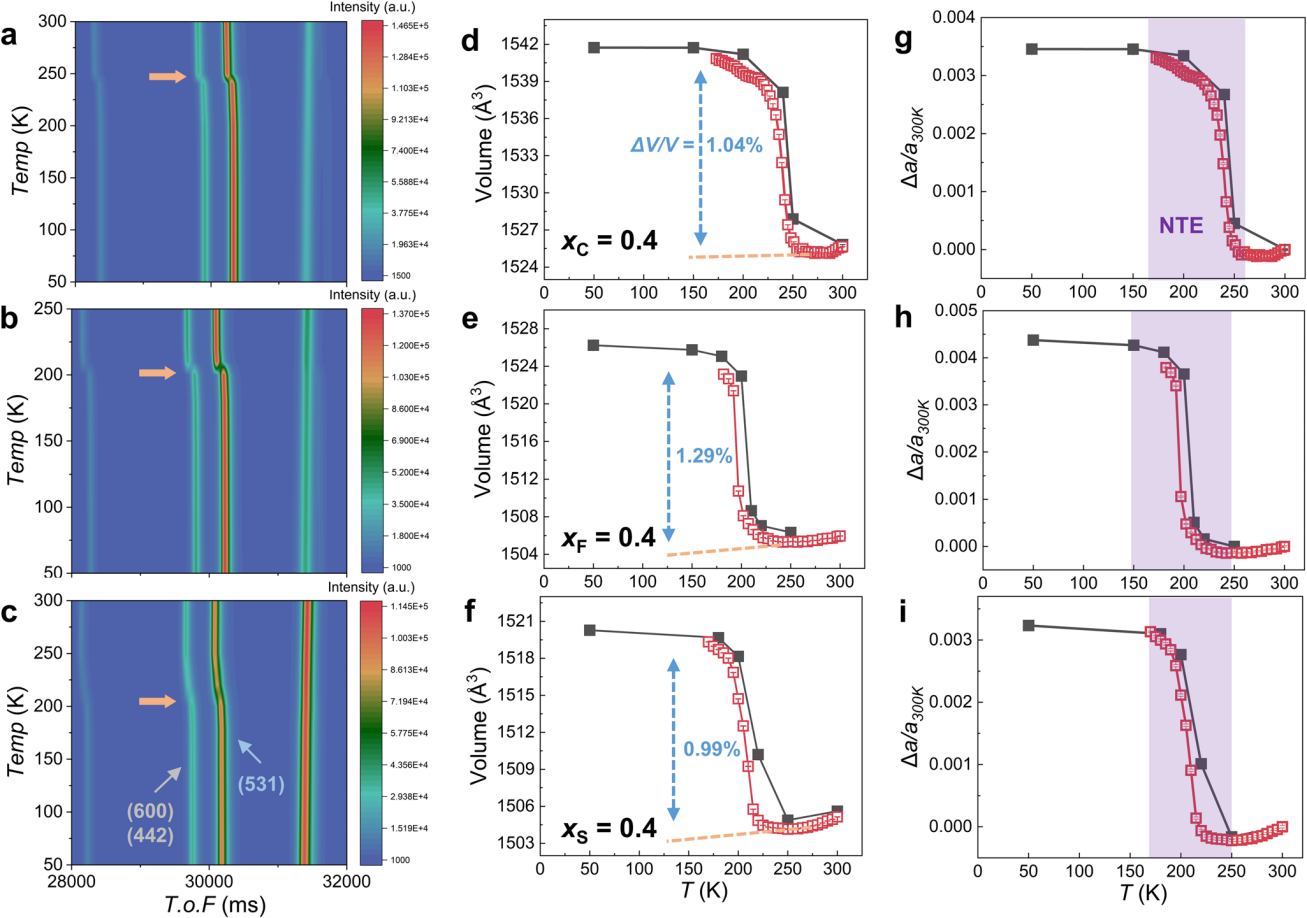
Contour plots of the temperature‐dependent ND patterns for the a) LaFe_11.6_Si_1.4_C_0.4_, b) LaFe_11.6_Si_1.4_F_0.4_, c) LaFe_11.6_Si_1.4_S_0.4_ samples. d–f) Corresponding thermal evolution of *V* extracted from ND (black curves) and synchrotron XRD (red open curves) for the above samples. g–i) Corresponding linear thermal expansion Δ*a/a_300K_* extracted from ND (black curves) and synchrotron XRD (red open curves) for the selected samples. The NLTE has been marked in the light purple area.

Moreover, compared with XRD, ND possesses a higher sensitivity to neighboring elements and light elements (e.g., H, B, C, N, O, F, S) due to strong variations in elemental scattering length. Therefore, it has been successfully employed to resolve the site occupation of external dopants within the lattice structure and magnetic moments and investigate the microstructural changes in atomic scales for different MCEMs, e.g. (Mn,Fe)_2_(P,Si)‐based, La(Fe,Si)_13_‐type, NiMn‐based Heusler, MnMX ferromagnet compounds.^[^ ^59^, ^93^, ^94^, ^95^ ^]^ Here, we present the in‐situ temperature‐dependent TOF ND data from 50 to 300 K for LaFe_11.6_Si_1.4_C_0.4_, LaFe_11.6_Si_1.4_F_0.4_, and LaFe_11.6_Si_1.4_S_0.4_ samples, as shown in **Figure** **6**a–c, respectively. It is noted that the characteristic diffraction peaks belonging to the (600), (442), and (531) planes show a clear discontinuity in the vicinity of *T_C_* (marked as a yellow arrow) for the *x_C_* = 0.4 and *x_F_* = 0.4 samples, which reflects its iso‐structural FOMT nature across the transition. One may notice that all peaks move to lower values during the FM‐PM transition upon heating, indicating that the unit cell contracts. However, in Figure 6c there is no distinct structural discontinuity for the *x_S_* = 0.4 sample, suggesting the SOMT nature with smaller lattice distortion across the transition. All the powder ND patterns have been refined and the corresponding lattice information has been summarized in Table S2 (Supporting Information). As demonstrated in Figure S7a–c (Supporting Information) good fit for the selected refined patterns as a function of the wave vector transfer *Q* was obtained using a cubic unit cell (space group Fm‐3c). Considering the potential interstitial sites *(24c, 24d, 48e, 48f, 64g, and 96h)*,^[^ ^96^ ^]^ the accurate preferred sites for the light element atoms have been obtained: i) C preferentially occupies the interstitial *24d* site, ii) F occupies the interstitial *24d* and substitutional *96i(Si)* sites, while S does not enter the main phase matrix. For the NaZn_13_‐type La(Fe,Si)_13_‐based compounds, it was found that *8a* and *8b* sites are distinctly occupied by La and Fe atoms, but the *96i* site is shared with Fe and Si atoms.^[^ ^97^ ^]^ Previous studies revealed that C atoms preferentially enter the interstitial *24d* site,^[^ ^91^, ^97^, ^98^ ^]^ which is consistent with our results. Furthermore, the lattice volume *V* for the *x*_C_ = 0.4, *x*_F_ = 0.4, and *x*_S_ = 0.4 alloys, derived from ND experiments, are shown in Figure 6d–f, respectively. It is found that the *V* exhibits discontinuous changes depending on their magnetic states with anomalies in the order of 1.04, 1.29, and 0.99%, while the lattice symmetry is conserved. The discontinuity in *V* also confirms the FOMT character for these four compositions, where a larger *V* change always contributes to an enhanced |Δ*s_m_* |, as shown in Figure 2.

Additionally, temperature‐dependent synchrotron HE‐XRD measurements are applied to these samples. The good refinements in Figure S8a–d (Supporting Information) indicate the following phase compositions: i) for parent compound with *α‐Fe* (6.2(2) wt.%; space group: Im‐3 m), ii) for *x_C_* = 0.4 with *α‐Fe* (3.3(2) wt.%) and LaFeSi (0.6(1) wt.%; space group: P4/nmm), iii) for *x*_F_ = 0.4 with *α‐Fe* (6.2(1) wt.%), LaFeSi (0.2(1) wt.%) and La(Fe,F)_2_ (2.7(1) wt.%; space group: Fm‐3 m), and iv) for *x*_S_ = 0.4 with *α‐Fe* (15.1(2) wt.%) and LaS (5.7(1) wt.%; space group: Fm‐3 m). The quantitative comparison of phase fractions determined from lab XRD, HE‐XRD, ND, and magnetic measurements has been shown in Table S3 (Supporting Information) for the selected parent compound, *x*_C_ = 0.4, *x*_F_ = 0.4, and *x*_S_ = 0.4 samples. For the S‐modified materials the significantly reduced *|Δs_m_|* value should be ascribed to the dramatically improved concentration of concomitant impurities, where increasing impurity levels (*α*‐Fe and LaS) have been found for increasing dopant contents, as demonstrated in Figure S9 (Supporting Information). In Figure 6d–f, the *V* changes obtained from synchrotron HE‐XRD (red open curves) and ND are close to each other. The refinement results at different temperatures have been collected in Tables S4–S7 (Supporting Information). Based on the Debye‐Grüneisen model,^[^ ^99^ ^]^ the magnetoelastic coupling can be evaluated by: Δ*V*  =  *V* −  *V_nm_* =  *k* × |*M* ^2^| + Δ*V_i_*. Here Δ*V* reflects the magnetic contribution, which corresponds to the difference between experimental *V* and the non‐magnetic unit cell volume *V_nm_*, *k* is the coupling constant, *M* stands for the magnetic moments, Δ*V_i_* results from the local magnetic moment and spin fluctuations.^[^ ^100^, ^101^ ^]^ As present in Figure S10a–d (Supporting Information), the Δ*V* values for *x*_C_ = 0.4, *x*_F_ = 0.4 and *x*_S_ = 0.4 samples have been extracted. Furthermore, in Figure S10d (Supporting Information) the almost linear relationship between Δ*V* and *M* ^2^ reveals that there is a magnetoelastic coupling among these materials,^[^ ^102^, ^103^ ^]^ and the experimental slopes indicate that the S‐doped material has a weaker magnetoelastic coupling compared with the C and F doped materials. It is noteworthy that this sample that hosts a SOMT nature, the magnetoelastic coupling still exists, and the phenomenon was also authenticated in some other SOMT materials such as AlFe_2_B_2_,^[^ ^104^, ^105^ ^]^ Mn_2_Sb^[^ ^106^ ^]^ and Laves phase systems.^[^ ^107^ ^]^ Moreover, as illustrated in Figure 6g–i, the magnetoelastic coupling results in a negative thermal lattice expansion (NTLE) for a certain temperature range, manifesting itself in the Δ*a/a_300K_* values for these four samples. The average thermal expansion coefficients (TECs) have been further calculated by.^[^ ^108^ ^]^ The average TECs for *x*_C_ = 0.4, *x*_F_ = 0.4 and *x*_S_ = 0.4 samples are −34.65 × 10^−6^ K^−1^, −42.68 × 10^−6^ K^−1^ and −41.94 × 10^−6^ K^−1^, which are comparable to other La(Fe,Si)_13_‐based derivatives.^[^ ^24^, ^108^, ^109^ ^]^

**Figure 7:**
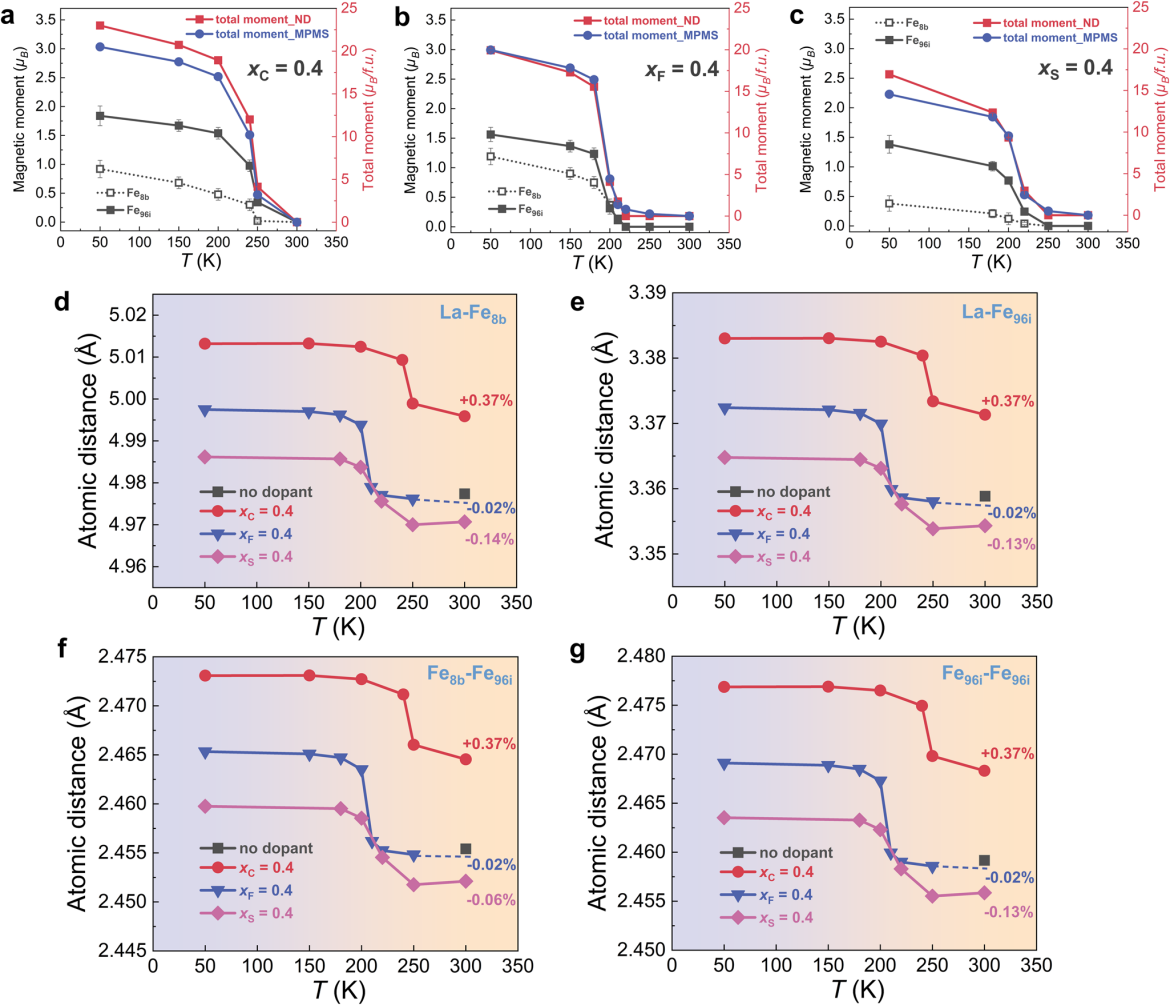
Experimental magnetic moments obtained from ND and SQUID magnetization measurements as a function of temperature for a) LaFe_11.6_Si_1.4_C_0.4_, b) LaFe_11.6_Si_1.4_F_0.4_, c) LaFe_11.6_Si_1.4_S_0.4_ samples. The total moment is listed on the right *y*‐axis. Corresponding interatomic distances of d) *La‐Fe_8b_, e) La‐Fe_96i_, f) Fe_8b_‐Fe_96i_, g) Fe_96i_‐Fe_96i_* as a function of temperature for the above materials, determined from ND.

Furthermore, because of the neutron spin, ND is sensitive to not only the lattice structure but also to the magnetic properties.^[^ ^7^ ^]^ For example, as present in Figure S10e (Supporting Information) one can distinguish the FM contribution for different samples among the FM state/two‐phase coexistence/PM state, determined from ND. The magnetic moments of the Fe atoms at different temperatures have been extracted and are presented in **Figure** **7**a–c for the *x*_C_ = 0.4, *x*_F_ = 0.4, and *x*_S_ = 0.4 samples, respectively. It is found that the total moment derived from ND and MPMS measurements are in good agreement and follow the same trends. Note that the total moment from MPMS includes subtracts the contributions from *α*‐Fe (the net magnetization at 300 K (PM)). For La(Fe,Al/Si)_13_ compounds, it is well‐known that the Fe_I_ (8*b*) and Fe_II_ (96*i*) atoms are responsible for the total magnetic moment.^[^ ^96^, ^110^ ^]^ In Figure 7a–c it is observed that the saturated moments of Fe_96_ *_i_* ($m_{Fe_{96i}}$) are around 1.5–2.0 *μ*_B_/f.u. and Fe_8_ *_b_* ($m_{Fe_{8b}}$) are around 0.5–1.3 *μ*_B_/f.u. for C and F doped compounds, while for the *x*_S_ = 0.4 sample the $m_{Fe_{96i}}$ and $m_{Fe_{8b}}$ values are significantly reduced to 1.5 and 0.5 *μ*_B_/f.u. due to the increased impurities. The magnitude of the $m_{Fe_{96i}}$ and $m_{Fe_{8b}}$ is similar to previous studies.^[^ ^87^, ^90^, ^96^ ^]^ Moreover, different from other magnetoelastic coupled (Mn,Fe)_2_(P,Si) based materials, where the moment of Mn shows robustness during the FM‐PM transition,^[^ ^111^ ^]^ both the $m_{Fe_{96i}}$ and $m_{Fe_{8b}}$ continuously decrease towards the transition. To further understand the magnetic property changes, it has been found that subtle changes in atomic distances among various metallic‐metallic and metallic‐metalloid pairs are closely associated with the magnetic exchange interactions, which dominate the magnetoelastic coupling in MCEMs.^[^ ^59^, ^87^, ^104^ ^]^ As demonstrated in Figure 7d–g, for the *x*_C_ = 0.4, *x*_F_ = 0.4, and *x*_S_ = 0.4 materials the interatomic distances among different atomic pairs including *La‐Fe_8b_, La‐Fe_96i_, Fe_8b_‐Fe_96i_, and Fe_96i_‐Fe_96i_* as a function of temperature have been determined from ND. First, it is clearly noted that all atomic distances changed across the transition, and a sudden jump happened during the FM‐PM transition. Depending on the type of transition, for instance, the compound (*x*_S_ = 0.4) with a SOMT property presents a more gradual change of distances, in comparison to the other three samples with a FOMT. It should be mentioned that different lattice modification modes (substitutional or interstitial) could cause a different chemical pressure on the lattice expansion/contraction and therefore result in different magnetic properties.^[^ ^58^, ^59^ ^]^ For example, for La(Fe,Si)_13_‐based compounds, the interstitial H^[^ ^66^ ^]^ and C atoms can significantly modify the lattice and magnetic exchange interaction with a large *T_C_* shift, compared with the modest change in F doping. In this study, all samples show a positive shift in *T_C_* with increasing dopant content, which indicates an increase in magnetic exchange interaction.^[^ ^63^ ^]^ In Figure 7d–g, taking *T* = 300 K as an example, it is found that the interstitial C doping leads to the largest isotropic atomic distance changes (+0.37%) in all pairs, while the mixture entering F only slightly alters the distances (−0.02%). Note that the decreased atomic distances of different pairs for *x*_S_ = 0.4 could result from the modification of the chemical composition due to the increased impurities. In other words, the enhanced exchange interaction is closely related to the increased atomic distances between two Fe sites even the reduced Fe content in the main phase can function as well.^[^ ^112^, ^113^, ^114^ ^]^ For the rare‐earth (RE)‐Fe systems, it pointed out that the critical Fe‐Fe distance is ≈2.45Å, and strong FM could be induced by increasing the distance.^[^ ^115^ ^]^ Meanwhile, for instance, for similar Sm_2_Fe_17_ materials, the interstitial N atoms attributed to the lattice expansion accompanied by the improved *T_C_*.^[^ ^116^, ^117^ ^]^ Herein, it is concluded that for La(Fe,Si)_13_‐based compounds the strength of magnetic exchange could be proportional to the elongation among magnetic atomic pairs, combining the ND results for H‐inserted materials.^[^ ^91^ ^]^ In addition, intermetallic compounds naturally involve metallic and covalent bonding,^[^ ^118^ ^]^ especially for the itinerant‐electron metamagnetic (IEM) transition materials it has been elucidated that the transition is closely correlated with changes in the electronic structure.^[^ ^27^, ^100^, ^119^, ^120^ ^]^ For example, the *T_C_* modification of the Fe‐based RE‐Fe compounds upon doping had been connected with the spin‐fluctuation model because of the electronic structural changes.^[^ ^119^ ^]^ For the La(Fe,Si)_13_‐based materials, the itineracy of the *3d* Fe electrons is well noticed and it is found that the magnetism is intermediate between a full itineracy and full localization.^[^ ^61^, ^121^ ^]^ Hydrogenation is one of the most efficient methods to regulate *T_C_* and maintain excellent GMCE. Theoretically, it is noticed that the robustness of first‐order IEM after H insertion results from the smaller changes for the electronic and magnetic structure (only chemical pressure), in comparison to other light atoms B, C, and N.^[^ ^61^ ^]^ Different from the simple chemical pressure picture, particularly worth mentioning is that the potential formation of the *p–d* hybridization between Fe and non‐metallic atoms will reshape the shallow free energy landscape and ultimately destroy the first‐order IEM.^[^ ^61^, ^122^ ^]^ For the current studies, it is observed that compared with F (high electronegativity) the interstitial C atoms with lower electronegativity cause more remarkable distance changes of different atomic pairs. This could be ascribed to the different ways the dopants can enter the main phase. The 24*d* interstitial site, is neighboring to Fe_96i_ sites and is located between the icosahedral clusters,^[^ ^96^ ^]^ and therefore it is much sensitive and pronounced to the distance between the icosahedral clusters.^[^ ^123^ ^]^ Nonetheless, even if F atoms have a stronger ability to form covalent bonding with Fe, the mixture of substitutional and interstitial ways decreases the fraction on 96*i* and 24*d* site, which further reduce the *p–d* hybridization between Fe and F. The formed *p–d* hybridization will generate the electron transfer from the *Fe d‐*band to the *p‐*band of C/ F and would be expected to fill the p‐band of C/ F, which can further enhance the splitting of the *Fe d* band and thus improve the magnetic moment of the Fe supplying the electrons.^[^ ^124^ ^]^ Simultaneously, the modest changes in the unit‐cell volume upon F doping only exert limited influence on the lattice “breathing” (expansion or contraction). Therefore, the strong first‐order IEM characteristic for F‐doped materials is well maintained without degradation, but with a moderate increment in *T_C_*, compared with the H‐doped case. The results indicate that the interplay between the anomalous pressure effect on the lattice and the covalent hybridization contributes to the tunable magnetoelastic coupling in the La(Fe,Si)_13_‐based compounds.

**Figure 8:**
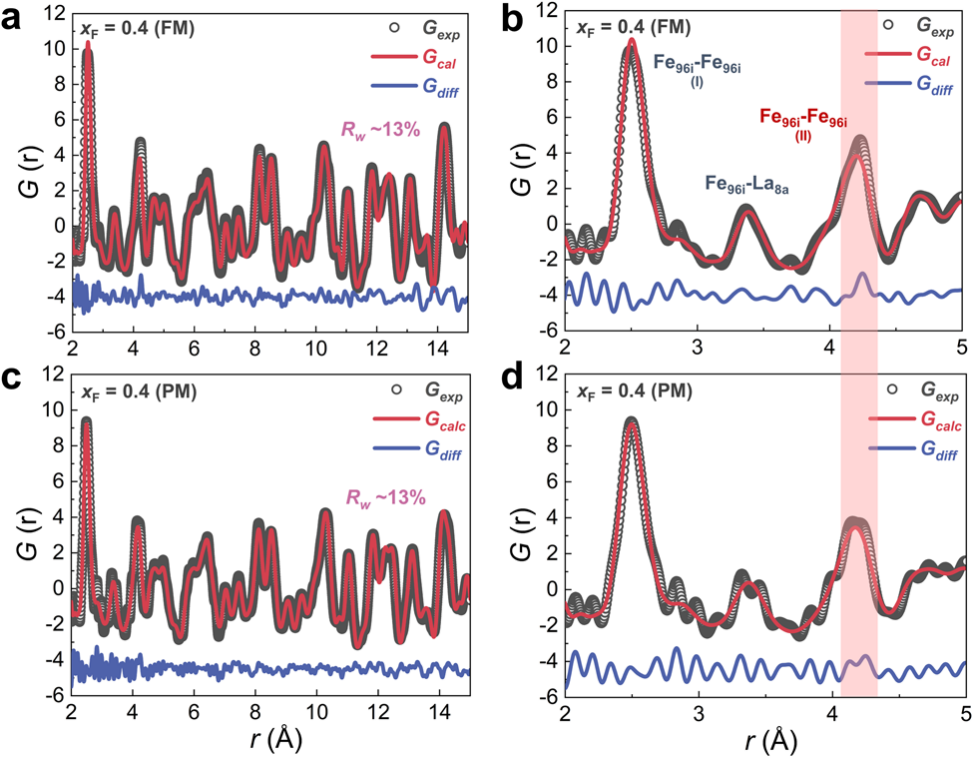
Experimental (open circle) and fitted (lines) PDF patterns for a,b) FM state at 150 K and c,d) PM state at 250 K in different *r* ranges for LaFe_11.6_Si_1.4_F_0.4_ sample. The difference curves between the observed and calculated PDF patterns are shown at the bottom.

To further reveal the impact of the local structure (including long‐range and short‐range structural correlations) and the lattice dynamics on the doped materials, a PDF analysis has been utilized, which enables quantitative refinement of the atomic structure on short length scales in real space in crystalline and amorphous materials including various MCEMs.^[^ ^7^, ^125^, ^126^ ^]^ The PDF analysis is carried out for the selected *x*_F_ = 0.4 sample below (150 K) and above (250 K) the phase transition. As present in Figure S11 (Supporting Information), the PDF *G*(*r*) patterns applying the cubic refinement model (same as the above conventional Rietveld refinement) have been determined by a fit to the ND data over the long‐range order of *r* = 15–45 Å. The refinement results yield a good fitting quality (e.g., *R_w_* values) for the *x*_F_ = 0.4 sample of both FM and PM phases. In **Figure** **8**a,b, the refined results of the PDF data are shown using the same cubic model for these two samples at FM and PM states, in which the low *r* region (2–15 Å) allows us to probe the local and intermediate structural changes. The reduced *R_w_* values for the four patterns (all below 13%) indicate that there is no obvious local structure distortion for the cubic lattice from long‐range order to short‐range order,^[^ ^127^, ^128^ ^]^ and the local structure is robust without local symmetry breaking for these FOMT materials. Furthermore, to capture local changes in different nearest neighboring atomic pairs before and after the transition, the corresponding zoom‐in patterns at lower *r* regions (2–5 Å) are also obtained, as illustrated in Figure 8c,d. From left to right, the three pronounced PDF peaks are recognized as Fe_96_ *_i_*‐Fe_96_ *_i_* (I), Fe_96_ *_i_*‐La_8_ *_b_*, and Fe_96_ *_i_*‐Fe_96_ *_i_* (II), respectively. It is known that there are in total five nearest Fe─Fe bonds (e.g., representing in the Fe_8_ *_b_* centered icosahedron structure^[^ ^97^ ^]^), and the current Fe_96_ *_i_*‐Fe_96_ *_i_* (I) peak belongs to the B_4_ Fe─Fe pair which is close to the 24*d* interstitial site.^[^ ^97^, ^117^ ^]^ Meanwhile, the Fe_96_ *_i_*‐Fe_96_ *_i_* (II) is one of the intra‐icosahedron Fe─Fe pairs. The schematic diagram of the atomic structure of the doped La(Fe,Si)_13_ compound in one unit cell has been demonstrated in Figure S12 (Supporting Information), as well as different types of Fe─Fe pairs. Additionally, the distance changes for the typical Fe_96_ *_i_*‐Fe_96_ *_i_* (I), Fe_96_ *_i_*‐La_8_ *_b_*, and Fe_96_ *_i_*‐Fe_96_ *_i_* (II) pairs are summarized in **Table** **2**, and it is observed the Fe_96_ *_i_*‐Fe_96_ *_i_* (I) only shows slight increase from FM to PM state. In contrast, Fe_96_ *_i_*‐La_8_ *_b_* has a negative movement after crossing the transition, and particularly for *x*_F_ = 0.4 samples the most significant decrease in Fe_96_ *_i_*‐Fe_96_ *_i_* (II) pair distance is observed with Δ *r_PM‐FM_* = −0.075 Å. The negative movement for Fe_96_ *_i_*‐La_8_ *_b_* and Fe_96_ *_i_*‐Fe_96_ *_i_* (II) is closely related to the NLTE phenomenon. The metastability of different atomic pairs further highlights the importance of tuning the micro‐environment of the strong magneto‐elastically coupled La(Fe,Si)_13_‐based compounds.

**Table 2:** Atomic distances in the FM state (Δ *r_FM_*) and PM state (Δ *r_PM_*) for different atomic pairs and distance differences between PM and FM states (Δ *r_PM‐FM_*) for LaFe_11.6_Si_1.4_F_0.4_ samples.

Sample	Atomic pairs	*r_FM_* [Å] [150 K]	*r_PM_* [Å] [250 K]	Δ *r_PM‐FM_* [Å]
	La_8_ *_a_*‐Fe_96_ *_i_*	3.3875	3.3725	−0.015
*x*_F_ = 0.4	Fe_96_ *_i_*‐Fe_96_ *_i_* (type I)	2.4875	2.4975	0.010
	Fe_96_ *_i_*‐Fe_96_ *_i_* (type II)	4.2275	4.1525	−0.075

## 3 Conclusions

In summary, different light elements (C, F, S) modified LaFe_11.6_Si_1.4_ compounds have successfully been synthesized, and their basic thermodynamic, magnetic, and microstructural properties have been investigated utilizing DSC, SQUID, FE‐EPMA, HR‐TEM, synchrotron HE‐XRD, ND, and PDF analysis. It is found that with increasing dopant contents all modified samples exhibit a higher *T_C_*, while with a negligible impact on the thermal hysteresis. The GMCE performance in C‐ and S‐modified samples with a higher content is significantly degraded, but it can be well maintained for the F‐doped samples. The preferential site occupancies of dopants are i) C – the interstitial 24*d* site, ii) F – the interstitial 24*d* and substitutional 96*i*(Si) sites, while S does not enter the matrix. The interstitial doping leads to more pronounced atomic distance changes and it is more efficient to shift *T_C_* compared with the mixed (substitutional/interstitial) doping, but the first‐order transition cannot be well kept because of different *p–d* hybridization degrees between Fe and dopants. Across the FOMT, there is no local structure breaking observed. These findings highlight the importance of the interplay between the lattice pressure effect and the covalent hybridization for the controllable itinerant‐electron metamagnetic transition, which further deepens our understanding of the La(Fe,Si)_13_‐based MCEMs and enhances the prospects for future applications.

## 4 Experimental Section

High‐purity (> 99.9%) raw materials including La pieces, Fe pieces, Si pieces, graphite powder, Fe_3_F powder, and sulfur powder are applied to prepare polycrystalline samples using high‐vacuum (< 10^−6^ mbar) arc‐melting under Ar atmosphere. The modified compounds correspond to LaFe_11.6_Si_1.4_C*_x_*_C_ (*x*_C_ = 0.0, 0.2, 0.4, 0.6, 0.8), LaFe_11.6_Si_1.4_F*_x_*_F_ (*x*_F_ = 0.0, 0.2, 0.4, 0.6) and LaFe_11.6_Si_1.4_S*_x_*_S_ (*x*_S_ = 0.0, 0.2, 0.4, 0.6, 0.8), respectively. The powder materials are pressed into pieces under ambient conditions for better melting reactions. The samples are melted 6 times for good homogeneity. To compensate for evaporation losses of La during melting, 5 at.% extra La is added. Subsequently, the as‐cast melted samples were sealed in quartz tubes under an Ar atmosphere (200 mbar) and annealed for 5 days at 1373 K in a vertical oven, followed by rapid quenching from the annealing furnace into cold water.

Zero‐field differential scanning calorimetry (DSC) measurements were performed utilizing a commercial TA‐Q2000 DSC calorimeter with a scanning rate of 10 K/min. To test the stability, the multi‐cycled DSC measurements have been conducted for the selected *x*_F_ = 0.4 sample, and the sequence follows i) before warming up (1st and 2nd cycle between 183–213 K), ii) warm up to 373 K and cool down to 183 K (3^rd^ cycle), iii) 4th and 5th cycle between 183–213 K. The iso‐field temperature‐dependent magnetization (*M–T*) and the iso‐thermal field‐dependent magnetization (*M–H*) curves for all samples were measured in a superconducting quantum interference device (SQUID, Quantum Design MPMS 5XL) magnetometer. The *M–H* measurements at different temperatures were performed by the so‐called loop method.^[^ ^129^ ^]^ The loose powder materials are selected for magnetic measurements, assuming a demagnetization factor of *N_d_* = 1/3 (assuming an equiaxed powder sample volume). Powder XRD measurements for all samples at RT in the paramagnetic (PM) state were carried out in a PANalytical *X*‐pert Pro diffractometer with Cu *K_α_* radiation. Temperature‐dependent synchrotron high‐energy XRD (HE‐XRD) measurements were performed on selected samples (including LaFe_11.6_Si_1.4_C_0.4_, LaFe_11.6_Si_1.4_F_0.4_ and LaFe_11.6_Si_1.4_S_0.4_) at the high‐energy synchrotron diffraction beamlines P21.1 and P21.2 at the PETRA‐III, DESY, Germany. The incident energy of the X‐ray beam was *E_photon_* = 100 keV (wavelength *λ* ≈ 0.123984 Å). During the measurements, the powder samples were mounted in Kapton capillaries, and the data were obtained in transmission geometry. In addition, temperature‐dependent ND experiments for the same samples as synchrotron HE‐XRD measurements were carried out on the time‐of‐flight (TOF) powder diffractometer at the BL16 Multi‐Physics Instrument (MPI) of the China Spallation Neutron Source (CSNS). More instrumental details can be found.^[^ ^130^ ^]^ About 6 g of powder sample was placed into a vanadium can (8 mm diameter) and neutron powder diffraction patterns were collected from the low‐temperature (50 K) ferromagnetic (FM) state to the high‐temperature (300 K) paramagnetic (PM) state. Neutron total scattering measurements for the LaFe_11.6_Si_1.4_F_0.4_ sample were also carried out below (150 K) and above (250 K) the phase transition temperature.^[^ ^131^ ^]^ The obtained XRD and ND patterns were analyzed using Fullprof's implementation of the Rietveld refinement method.^[^ ^132^ ^]^ The error bars for the refinement results have been included. The fitting and refinement for the pair distribution function (PDF) were executed using the PDFGui program.^[^ ^133^ ^]^ In addition, to study morphology and composition distribution, EPMA measurements for the selected samples were conducted using a JEOL JXA‐iHP200F field‐emission EPMA (FE‐EPMA) equipped with scanning electron microscopy (SEM) mode and energy dispersive X‐ray spectroscopy (EDS). The used acceleration voltage is 15 kV and the diameter of the electron beam for EPMA is ≈1 µm. The chemical compositions in at.% for the selected samples have been determined as La_6.6_Fe_78.6_Si_9.8_C_5.0_, La_8.6_Fe_82.7_Si_6.4_F_2.3_, and La_5.8_Fe_82.5_Si_11.7_, respectively. Note that there is an overestimation of C and the statistical errors (1–5%) should be considered. Moreover, transmission electron microscopy (TEM) specimens were prepared using the ion‐beam thinning method. HR‐TEM with EDS mapping measurements for the selected samples were performed using a JEM‐F200 instrument.

## Conflict of Interest

The authors declare no conflict of interest.

## Supporting information



Supporting Information

## Data Availability

The data that support the findings of this study are available from the corresponding author upon reasonable request.
