# Supplementary material for: Engineering Light‐Element Modified LaFe11.6Si1.4 Compounds Enables Tunable Giant Magnetocaloric Effect
Source: Adv Sci (Weinh). 2025 May 19;12(22):2416288. doi: 10.1002/advs.202416288 (PMC12165023; doi:10.1002/advs.202416288)
Supplement: Supplementary file 1 — Supporting Information [file ADVS-12-2416288-s001.docx]

**Supporting Information**

**Engineering Light-Element Modified LaFe_11.6_Si_1.4_ Compounds Enables Tunable Giant Magnetocaloric Effect**

Fengqi Zhang^1,2, *^, Ziying Wu^2^, Xiaofang Zhang^3^, Xiang Chi^3^, Zhenduo Wu^4^, Jianrong Gao^5^, Huaican Chen^6,7^, Wen Yin^6,7^, Ulrich Lienert^8^, Ann-Christin Dippel^8^, Martin v. Zimmermann^8^, Niels van Dijk^2^, Ekkes Brück^2, *^, Yang Ren^1,9, *^

Dr. Fengqi Zhang, Prof. Yang Ren

1. JC STEM Lab of Energy and Materials Physics, Department of Physics, City University of Hong Kong, Kowloon, Hong Kong SAR.

E-mail: [fzhan7@cityu.edu.hk](mailto:fzhan7@cityu.edu.hk) ; [yangren@cityu.edu.hk](mailto:yangren@cityu.edu.hk)

Dr. Fengqi Zhang, Ziying Wu, Dr. Niels van Dijk, Prof. Ekkes Brück

2. Fundamental Aspects of Materials and Energy (FAME), Faculty of Applied Sciences, Delft University of Technology, Mekelweg 15, 2629JB Delft, The Netherlands.

E-mail: [E.H.Bruck@tudelft.nl](mailto:E.H.Bruck@tudelft.nl)

Xiaofang Zhang, Xiang Chi

3. Songshan Lake Materials Laboratory, Dongguan, China.

Dr. Zhenduo Wu

4. City University of Hong Kong (Dongguan), Dongguan, China.

Prof. Jianrong Gao

5. Key Laboratory of Electromagnetic Processing of Materials (Ministry of Education), Northeastern University, Shenyang 110819, China.

Dr. Huaican Chen, Dr. Wen Yin

6. Spallation Neutron Source Science Center, Dalang, Dongguan 523803, China.

7. Institute of High Energy Physics, Chinese Academy of Sciences, Beijing 100049, China.

Dr. Ulrich Lienert, Dr. Ann-Christin Dippel, Dr. Martin v. Zimmermann

8. Deutsches Elektronen-Synchrotron DESY, Notkestraße 85, 22607 Hamburg, Germany.

Prof. Yang Ren

9. Center for Neutron Scattering, City University of Hong Kong, Kowloon, Hong Kong SAR.


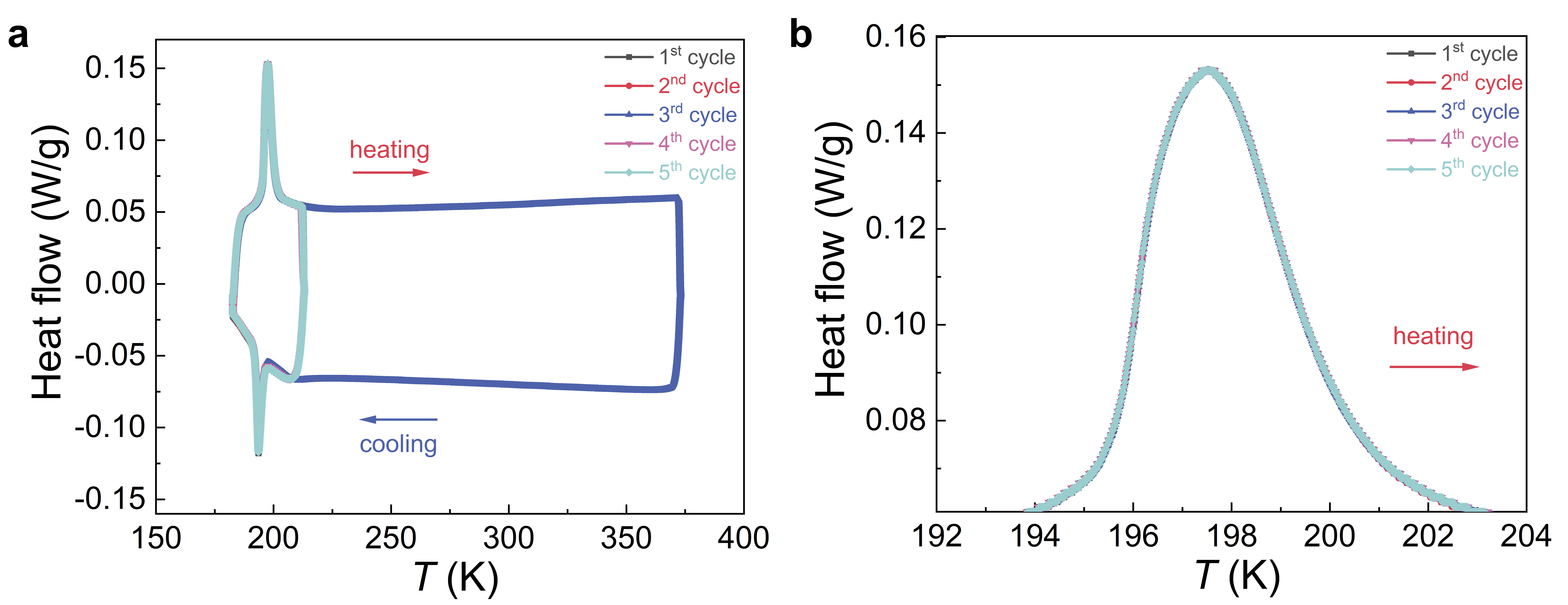


**Figure S1.** Multi-cycled DSC experiments upon warming and cooling processes at a rate of 10 K/min for *x*_F_ = 0.4 sample.


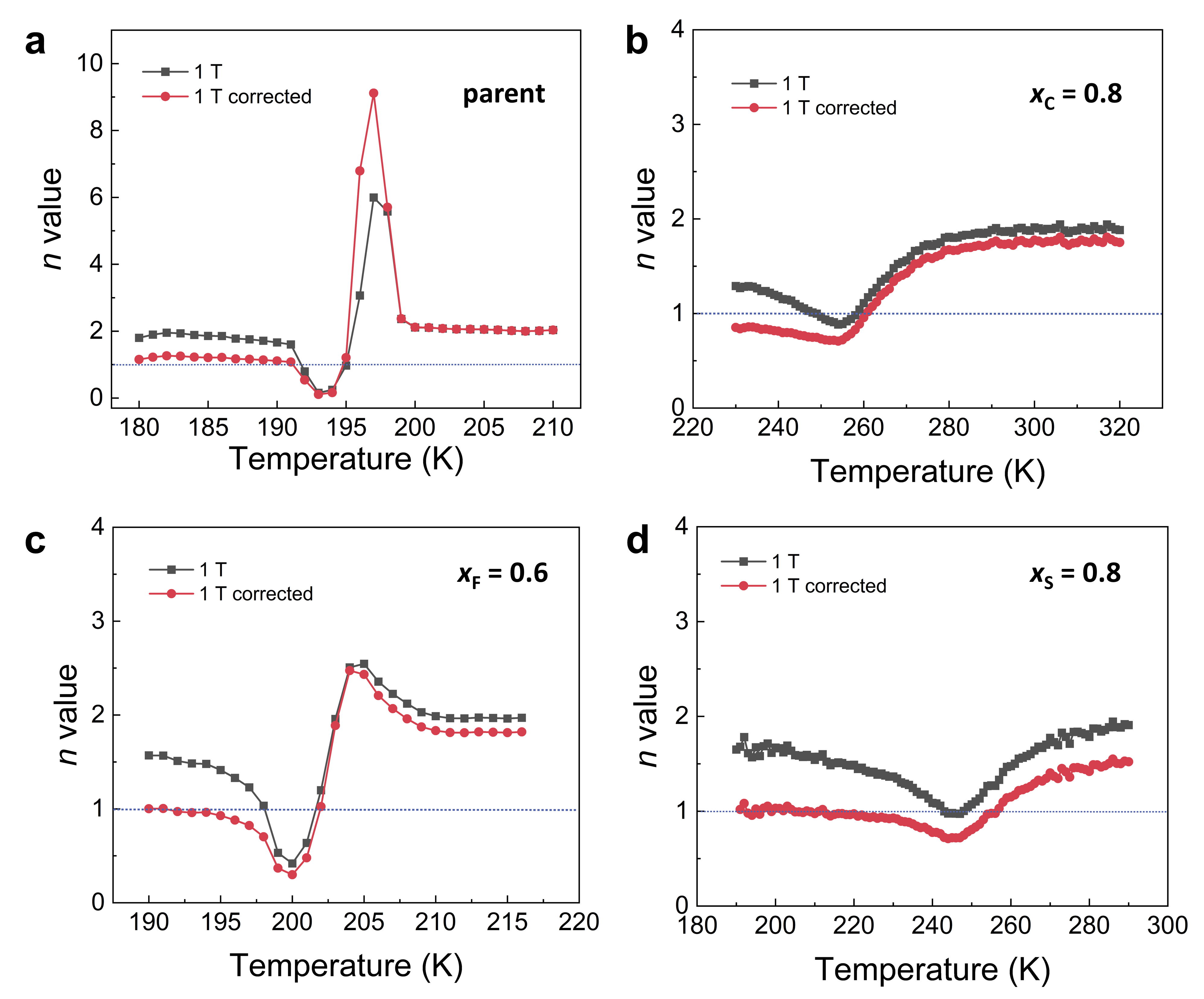


**Figure S2.** Obtained *n* values before and after calibration at 1 T for the selected samples. Note that the deviations of *n* values at PM state for C/F/S samples result from *α*-Fe impurities.


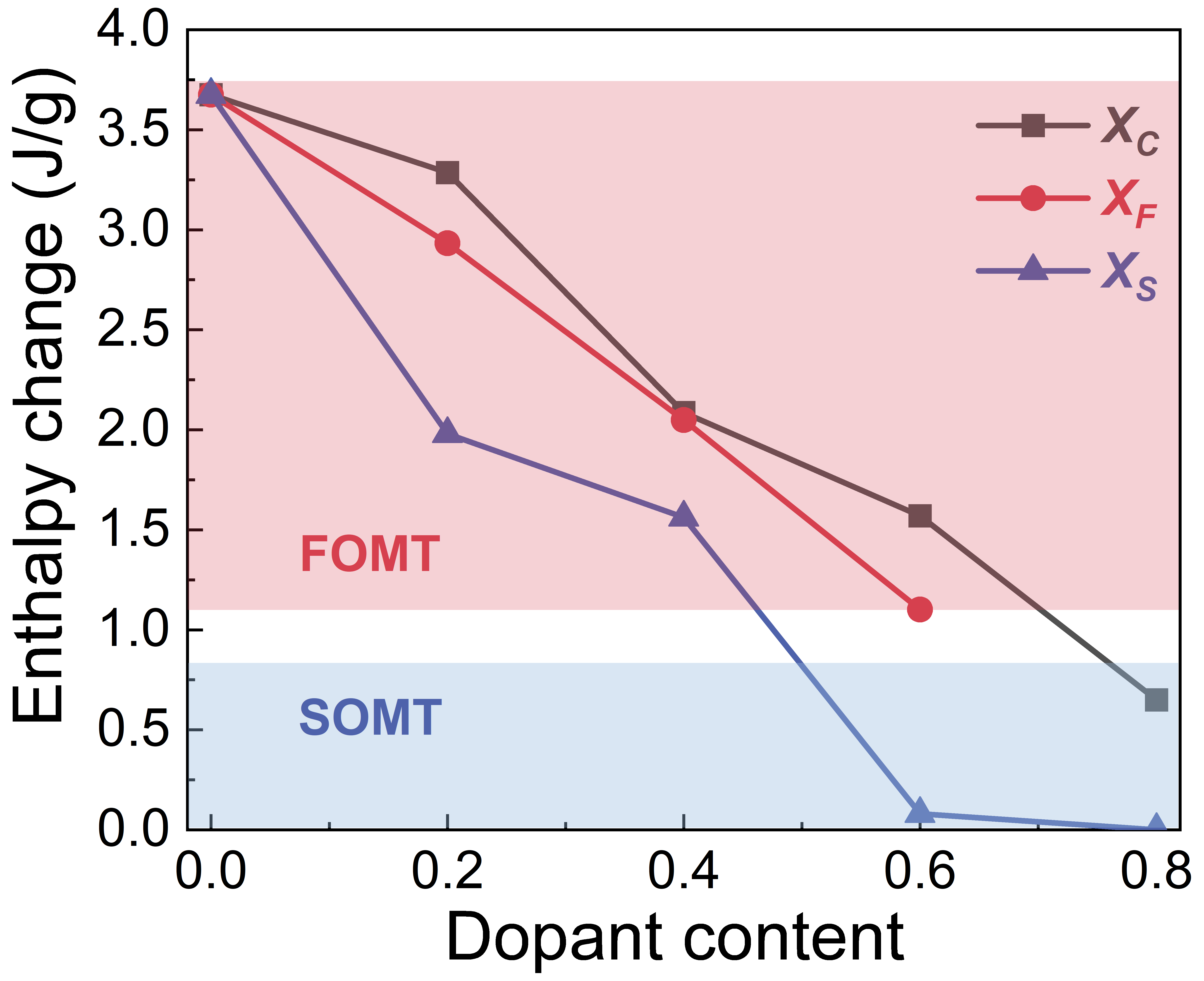


**Figure S3.** Enthalpy change derived from DSC experiments as a function of dopant content for LaFe_11.6_Si_1.4_C*_x_*_C_ (*x*_C_ = 0.0, 0.2, 0.4, 0.6, 0.8), LaFe_11.6_Si_1.4_F*_x_*_F_ (*x*_F_ = 0.0, 0.2, 0.4, 0.6) and LaFe_11.6_Si_1.4_S*_x_*_S_ (*x*_S_ = 0.0, 0.2, 0.4, 0.6, 0.8) samples.

**Table S1.** Summary of lattice parameter *a* for LaFe_11.6_Si_1.4_C*_xC_* (*x_C_* = 0.0, 0.2, 0.4, 0.6, 0.8), LaFe_11.6_Si_1.4_F*_xF_* (*x_F_* = 0.0, 0.2, 0.4, 0.6) and LaFe_11.6_Si_1.4_S*_xS_* (*x_S_* = 0.0, 0.2, 0.4, 0.6, 0.8) samples, extracted from lab-based XRD measurements at room temperature.

| **Dopant content** | ***a_C_* (Å)** | ***a_F_ (Å)*** | ***a_S_ (Å)*** |
| --- | --- | --- | --- |
| 0.0 | 11.4770(1) | 11.4770(1) | 11.4770(1) |
| 0.2 | 11.5004(1) | 11.4697(1) | 11.4712(1) |
| 0.4 | 11.5118(1) | 11.4701(1) | 11.4647(1) |
| 0.6 | 11.5294(2) | 11.4737(1) | 11.4612(1) |
| 0.8 | 11.5278(1) |  | 11.4560(1) |

**
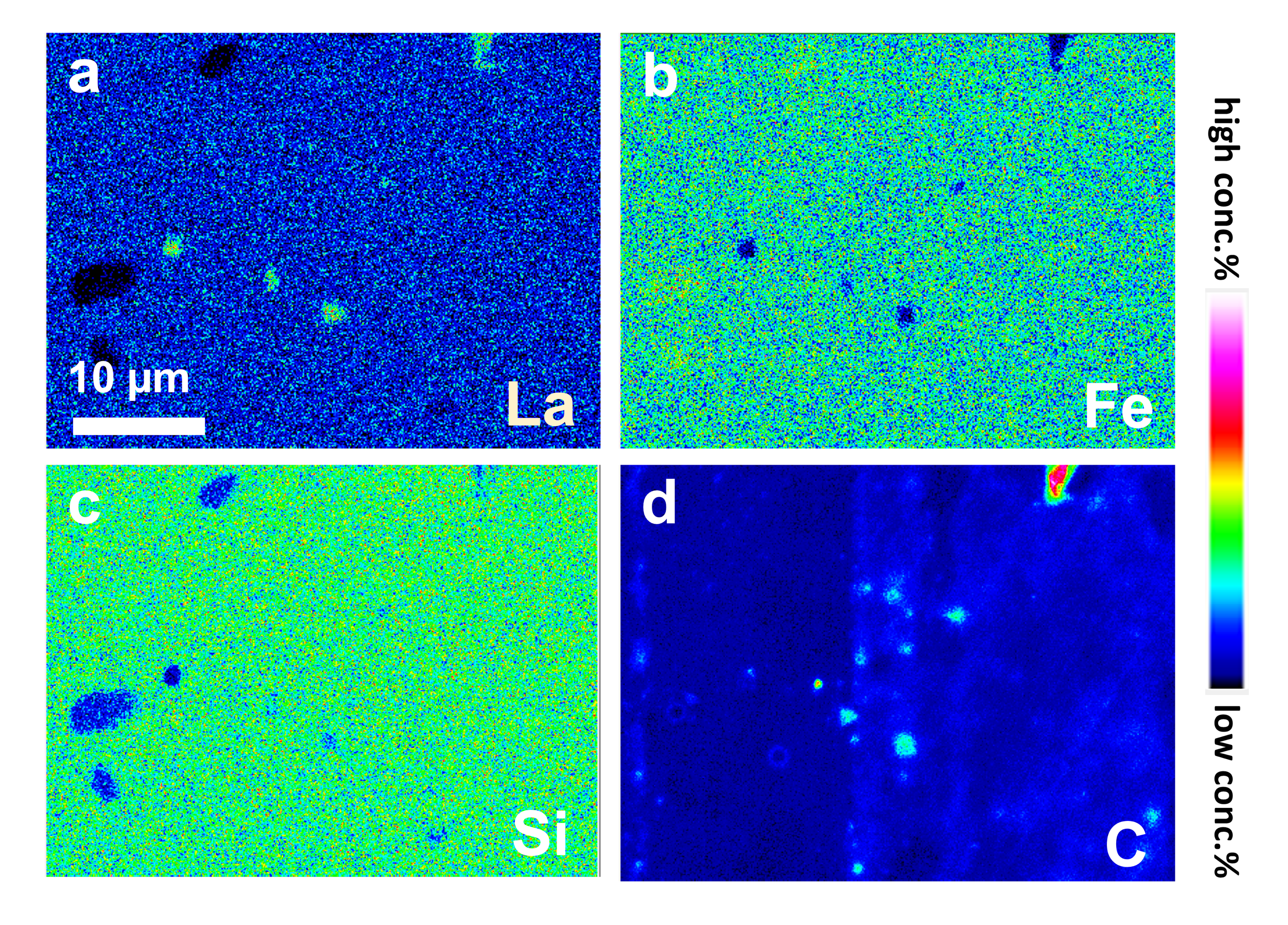
**

**Figure S4.** FE-EPMA elemental mapping images for (a) La, (b) Fe, (c) Si, (d) C elements for LaFe_11.6_Si_1.4_C_0.4_ samples.

**
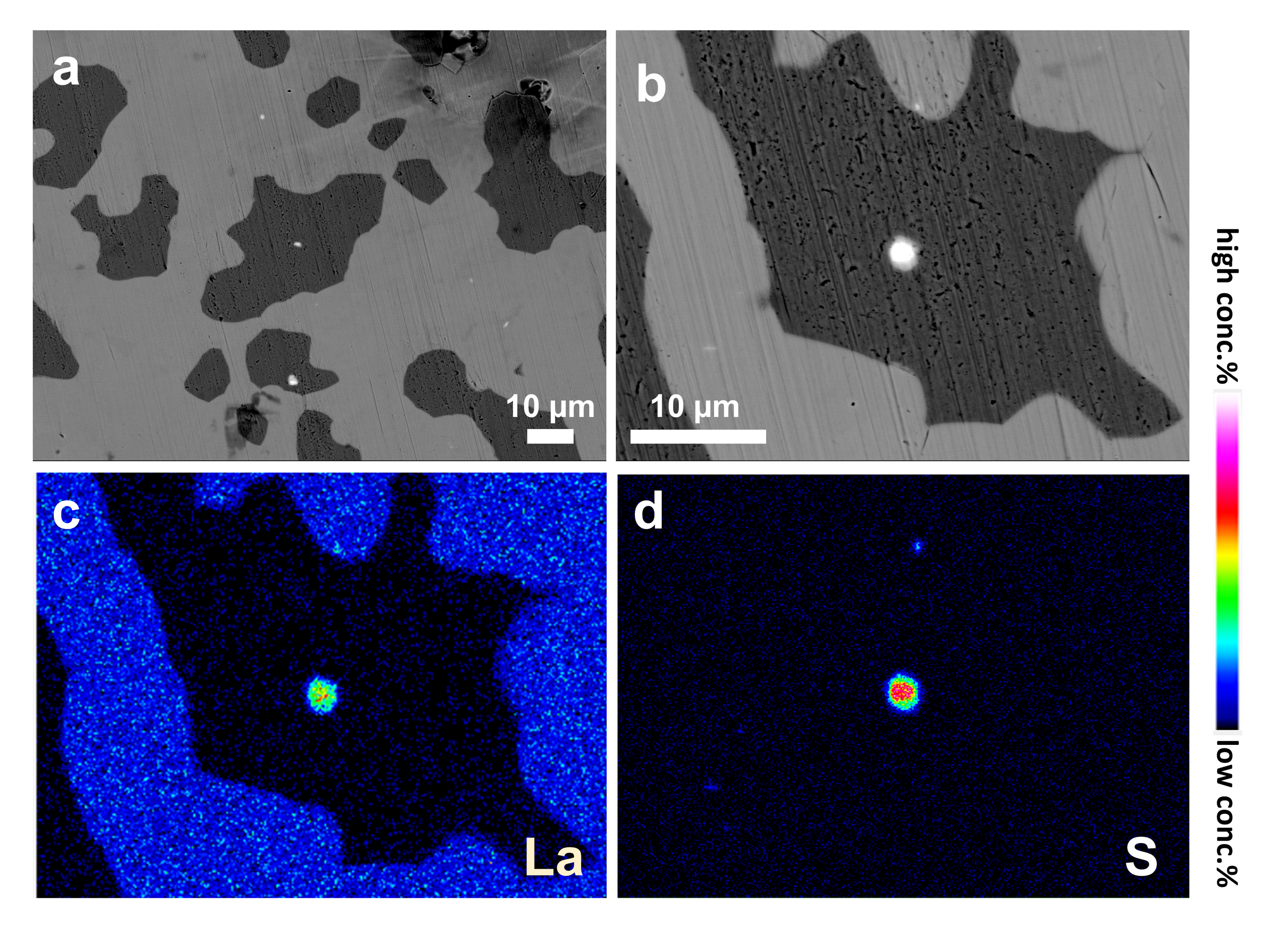
**

**Figure S5.** (a) Back-scattered SEM image for LaFe_11.6_Si_1.4_S_0.4_ samples. The light grey area stands for main phase. (b) Back-scattered SEM image in different region for the same sample for the FE-EPMA elemental mapping images for (c) La and (d) S elements.


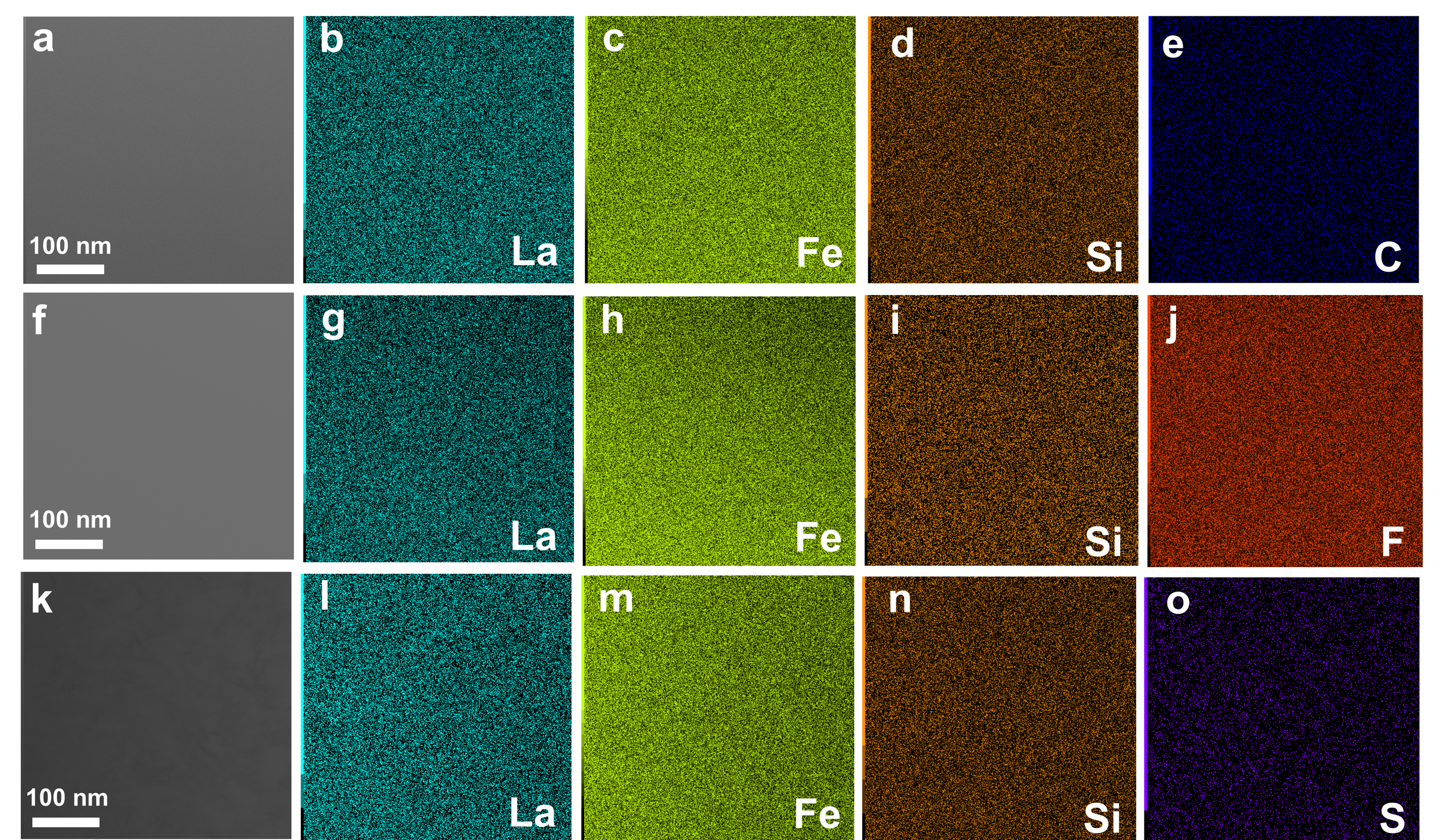


**Figure S6.** (a) HR-TEM image of the main phase for C = 0.4 sample. (b-e) Corresponding elemental mapping images of La, Fe, Si and C, respectively. (f) HR-TEM image of the main phase for *x*_F_ = 0.4 sample. (g-j) Corresponding elemental mapping images of La, Fe, Si and F, respectively. (k) HR-TEM image of the main phase for *x*_S_ = 0.4 sample. (l-o) Corresponding elemental mapping images of La, Fe, Si and S, respectively.


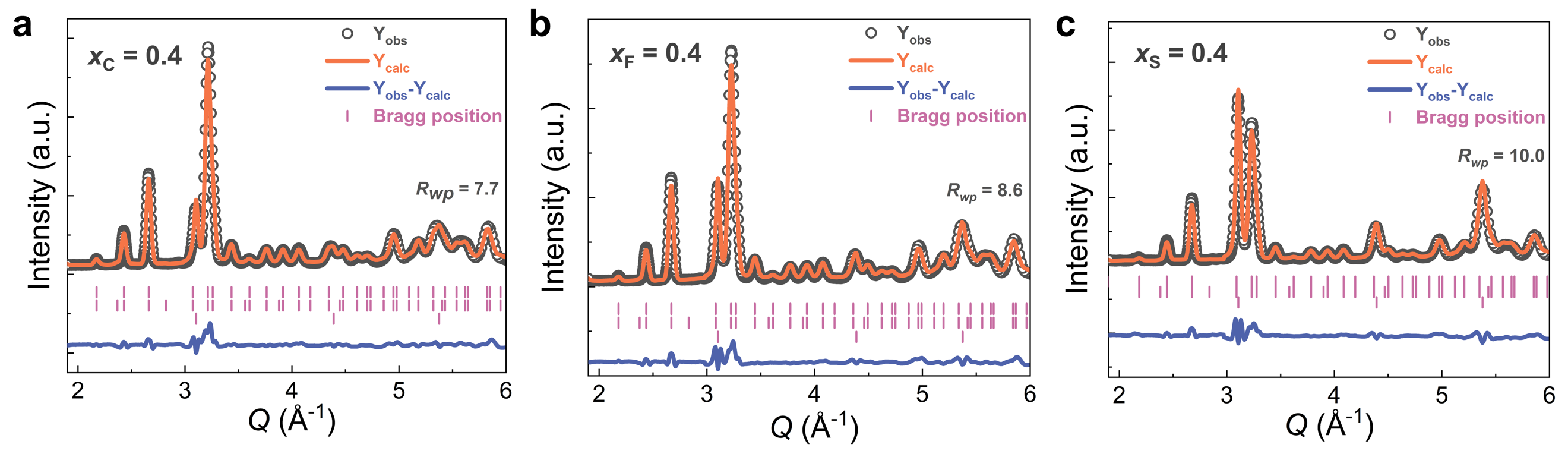


**Figure S7.** Fitted powder neutron diffraction patterns as a function of the wave vector transfer *Q* at 50 K (FM state) for (a) *x*_C_ = 0.4 (b) *x*_F_ = 0.4 and (c) *x*_S_ = 0.4 samples, collected from detector bank 4 at the MPI instrument. Black circles indicate the observed data points, red lines the calculated profile, blue lines the residuals and vertical lines the Bragg peak positions.

**Table S2.** Summary of lattice parameter *a*, magnetic moments of different magnetic atoms, total moments from ND and SQUID measurements, and atomic distances in La-Fe_8_*_b_*, La-Fe_96_*_i_*, Fe_96_*_i_*-Fe_96_*_i_* and Fe_8_*_b_*-Fe_96_*_i_* pairs for LaFe_11.6_Si_1.4_C_0.4_, LaFe_11.6_Si_1.4_F_0.4_ and LaFe_11.6_Si_1.4_S_0.4_ samples, extracted from temperature-dependent ND measurements.


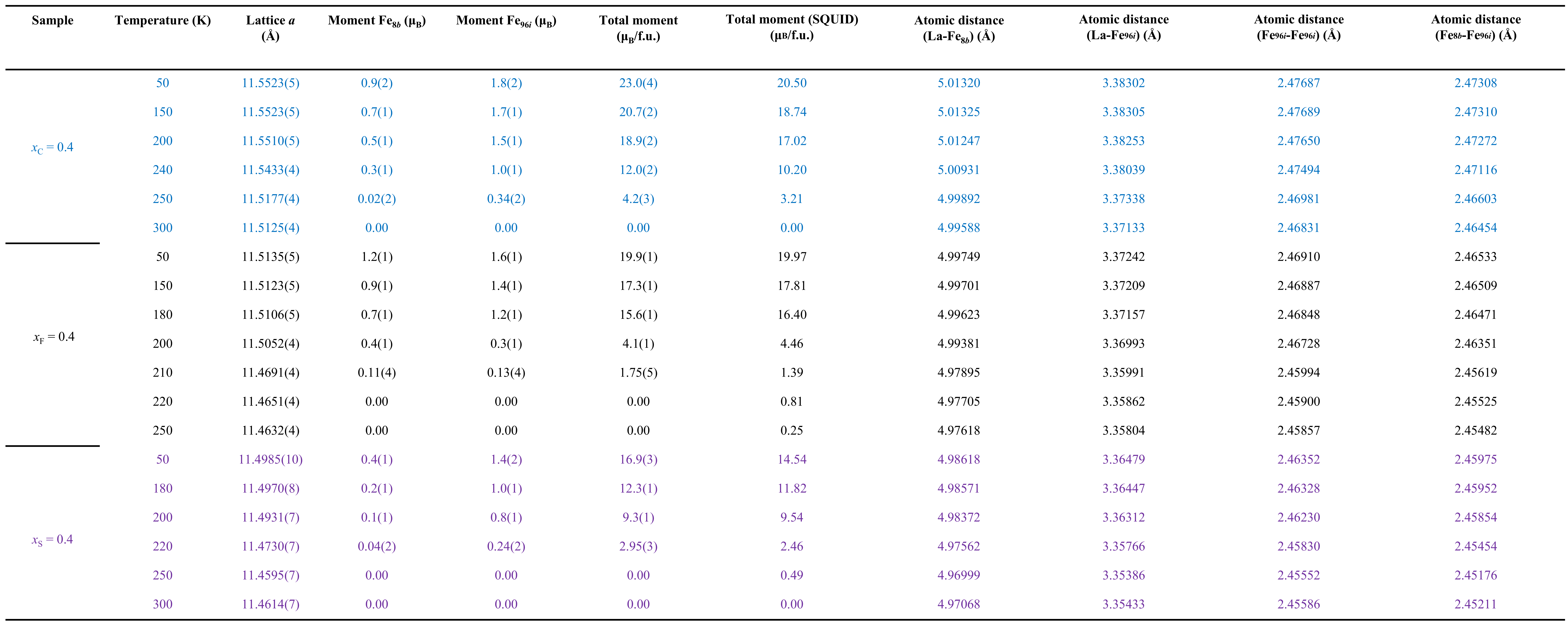


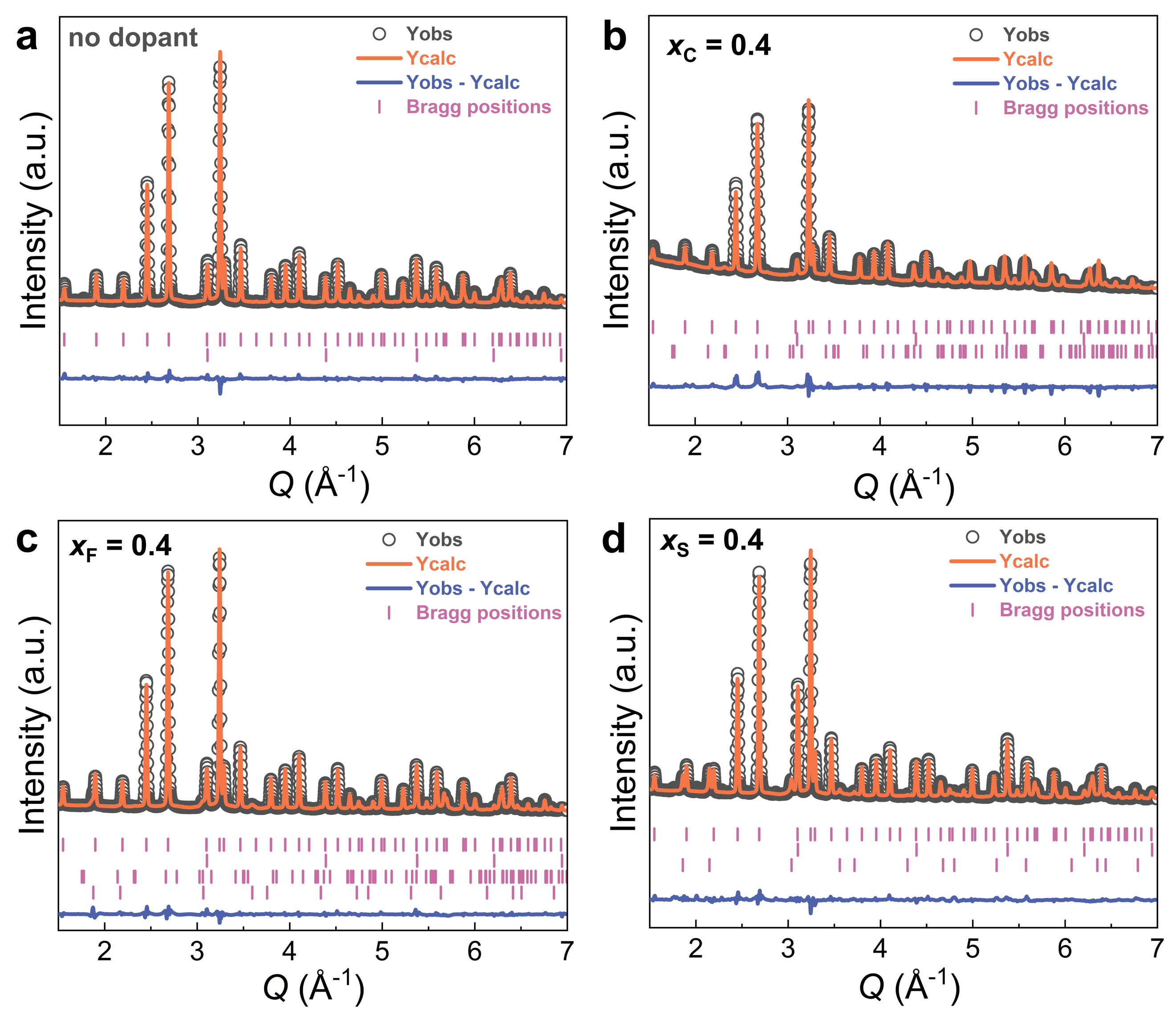


**Figure S8.** Fitted synchrotron HE-XRD patterns as a function of the wave vector transfer *Q* at 300 K for (a) no dopant (b) *x*_C_ = 0.4 (c) *x*_F_ = 0.4 and (d) *x*_S_ = 0.4 samples. Black circles indicate the observed data points, red lines the calculated profile, blue lines the residuals and vertical lines the Bragg peak positions. Black circles indicate the observed data points, orange lines the calculated profile, blue lines the difference and vertical lines the Bragg peak positions (top-bottom: a. main phase and *α*-Fe impurity; b. main phase, *α*-Fe and LaFeSi based impurities; c. main phase, *α*-Fe, LaFeSi and La(Fe,F)_2_ based impurities; d. main phase, *α*-Fe and LaS based impurities).

**Table S3.** Quantitative comparison of phase fractions determined from lab XRD, HE-XRD, ND and magnetic measurements for the selected parent compound, *x_C_* = 0.4, *x_F_* = 0.4 and *x_S_* = 0.4 samples.


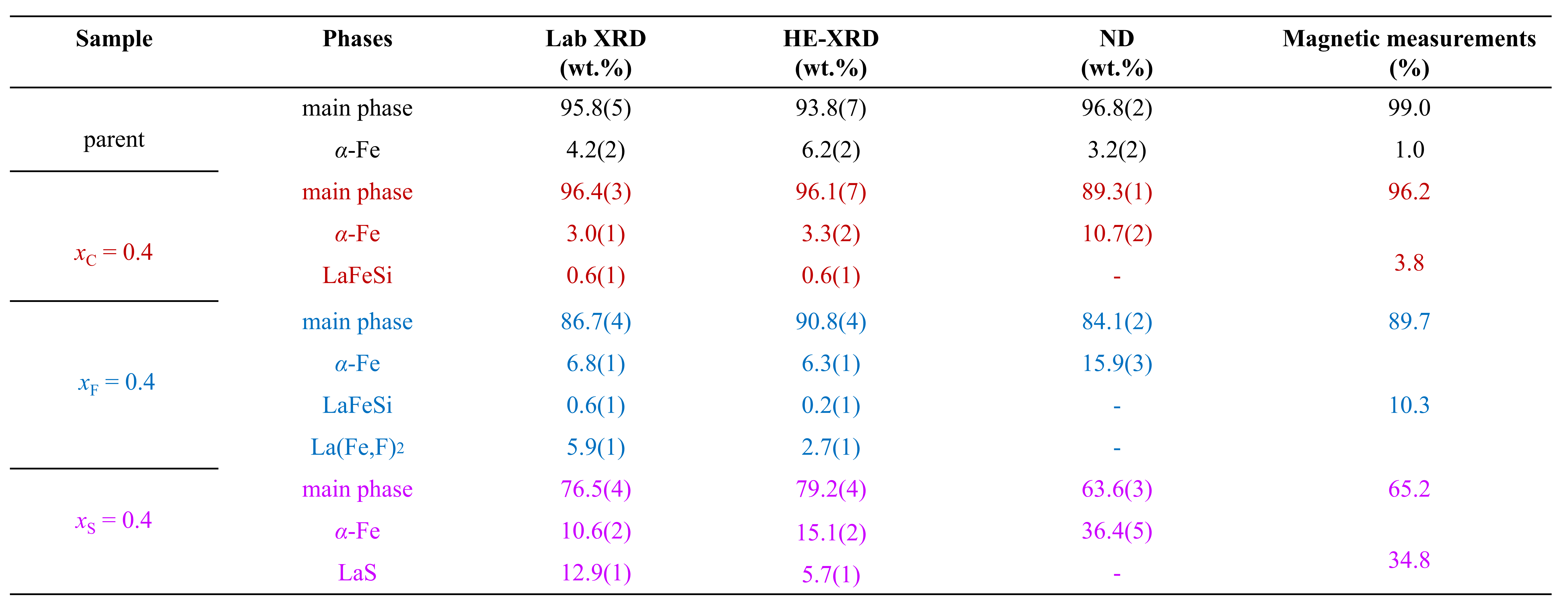


**Table S4.** Summary of lattice parameter *a* and concentration of different phases for parent LaFe_11.6_Si_1.4_ sample, extracted from synchrotron HE-XRD measurements at different temperatures.

| **Sample** | **Temperature (K)** | ***Lattice a* (Å)** | **Temperature (K)** | ***Lattice a* (Å)** | **Temperature (K)** | ***Lattice a* (Å)** | **Temperature (K)** | ***Lattice a* (Å)** | ***Main phase (wt.%)*** | ***α-Fe***  ***(wt.%)*** |
| --- | --- | --- | --- | --- | --- | --- | --- | --- | --- | --- |
| Parent compound | 176 | 11.5170(6) | 209 | 11.4655(6) | 242 | 11.4630(6) | 275 | 11.4658(6) | 93.8(7) | 6.2(2) |
|  | 179 | 11.5165(6) | 212 | 11.4644(6) | 245 | 11.4632(6) | 278 | 11.4662(6) |  |  |
|  | 182 | 11.5161(6) | 215 | 11.4637(6) | 248 | 11.4633(6) | 281 | 11.4665(6) |  |  |
|  | 185 | 11.5155(6) | 218 | 11.4634(6) | 251 | 11.4636(6) | 284 | 11.4669(6) |  |  |
|  | 188 | 11.5145(6) | 221 | 11.4631(6) | 254 | 11.4640(6) | 287 | 11.4673(6) |  |  |
|  | 191 | 11.5127(6) | 224 | 11.4629(6) | 257 | 11.4643(6) | 290 | 11.4677(6) |  |  |
|  | 194 | 11.5093(6) | 227 | 11.4629(6) | 260 | 11.4644(6) | 293 | 11.4681(6) |  |  |
|  | 197 | 11.4713(6) | 230 | 11.4628(6) | 263 | 11.4646(6) | 296 | 11.4684(6) |  |  |
|  | 200 | 11.4690(6) | 233 | 11.4628(6) | 266 | 11.4649(6) | 299 | 11.4685(6) |  |  |
|  | 203 | 11.4675(6) | 236 | 11.4630(6) | 269 | 11.4651(6) | 300 | 11.4687(6) |  |  |
|  | 206 | 11.4667(6) | 239 | 11.4630(6) | 272 | 11.4655(6) |  |  |  |  |

**Table S5.** Summary of lattice parameter *a* and concentration of different phases for LaFe_11.6_Si_1.4_C_0.4_ sample, extracted from synchrotron HE-XRD measurements at different temperatures.

| **Sample** | **Temperature (K)** | ***Lattice a* (Å)** | **Temperature (K)** | ***Lattice a* (Å)** | **Temperature (K)** | ***Lattice a* (Å)** | **Temperature (K)** | ***Lattice a* (Å)** | ***Main phase (wt.%)*** | ***α-Fe***  ***(wt.%)*** | ***LaFeSi***  ***(wt.%)*** |
| --- | --- | --- | --- | --- | --- | --- | --- | --- | --- | --- | --- |
| *x*_C_ = 0.4 | 173 | 11.5501(5) | 206 | 11.5465(6) | 239 | 11.5291(8) | 272 | 11.5107(9) | 96.1(7) | 3.3(2) | 0.6(1) |
|  | 176 | 11.5498(5) | 209 | 11.5463(6) | 242 | 11.5215(8) | 275 | 11.5110(9) |  |  |  |
|  | 179 | 11.5497(5) | 212 | 11.5462(6) | 245 | 11.5165(8) | 278 | 11.5107(10) |  |  |  |
|  | 182 | 11.5493(5) | 215 | 11.5460(7) | 248 | 11.5138(8) | 281 | 11.5108(10) |  |  |  |
|  | 185 | 11.5491(5) | 218 | 11.5455(7) | 251 | 11.5130(8) | 284 | 11.5108(10) |  |  |  |
|  | 188 | 11.5488(5) | 221 | 11.5449(7) | 254 | 11.5116(8) | 287 | 11.5107(10) |  |  |  |
|  | 191 | 11.5486(5) | 224 | 11.5437(7) | 257 | 11.5110(9) | 290 | 11.5110(10) |  |  |  |
|  | 194 | 11.5481(6) | 227 | 11.5426(7) | 260 | 11.5116(9) | 293 | 11.5114(10) |  |  |  |
|  | 197 | 11.5475(6) | 230 | 11.5410(7) | 263 | 11.5111(9) | 296 | 11.5118(11) |  |  |  |
|  | 200 | 11.5472(6) | 233 | 11.5388(7) | 266 | 11.5110(9) | 299 | 11.5119(11) |  |  |  |
|  | 203 | 11.5467(6) | 236 | 11.5348(8) | 269 | 11.5109(9) | 300 | 11.5121(11) |  |  |  |

**Table S6.** Summary of lattice parameter *a* and concentration of different phases for LaFe_11.6_Si_1.4_F_0.4_ sample, extracted from synchrotron HE-XRD measurements at different temperatures.

| **Sample** | **Temperature (K)** | ***Lattice a* (Å)** | **Temperature (K)** | ***Lattice a* (Å)** | **Temperature (K)** | ***Lattice a* (Å)** | ***Main phase (wt.%)*** | ***α-Fe***  ***(wt.%)*** | ***LaFeSi***  ***(wt.%)*** | ***La(FeF)_2_***  ***(wt.%)*** |
| --- | --- | --- | --- | --- | --- | --- | --- | --- | --- | --- |
| *x*_F_ = 0.4 | 182 | 11.5058(3) | 227 | 11.4616(3) | 272 | 11.4611(3) | 90.8(4) | 6.3(1) | 0.2(1) | 2.7(1) |
|  | 187 | 11.5045(3) | 232 | 11.4609(3) | 277 | 11.4611(3) |  |  |  |  |
|  | 192 | 11.5013(3) | 237 | 11.4608(3) | 282 | 11.4615(3) |  |  |  |  |
|  | 197 | 11.4744(3) | 242 | 11.4606(3) | 287 | 11.4616(3) |  |  |  |  |
|  | 202 | 11.4677(3) | 247 | 11.4607(3) | 292 | 11.4617(3) |  |  |  |  |
|  | 207 | 11.4656(3) | 252 | 11.4608(3) | 297 | 11.4621(3) |  |  |  |  |
|  | 212 | 11.4640(3) | 257 | 11.4606(3) | 300 | 11.4623(3) |  |  |  |  |
|  | 217 | 11.4627(3) | 262 | 11.4609(3) |  |  |  |  |  |  |
|  | 222 | 11.4618(3) | 267 | 11.4607(3) |  |  |  |  |  |  |

**Table S7.** Summary of lattice parameter *a* and concentration of different phases for LaFe_11.6_Si_1.4_S_0.4_ sample, extracted from synchrotron HE-XRD measurements at different temperatures.

| **Sample** | **Temperature (K)** | ***Lattice a* (Å)** | **Temperature (K)** | ***Lattice a* (Å)** | **Temperature (K)** | ***Lattice a* (Å)** | **Temperature (K)** | ***Lattice a* (Å)** | ***Main phase (wt.%)*** | ***α-Fe***  ***(wt.%)*** | ***LaS***  ***(wt.%)*** |
| --- | --- | --- | --- | --- | --- | --- | --- | --- | --- | --- | --- |
| *x*_S_ = 0.4 | 170 | 11.4961(2) | 205 | 11.4789(4) | 240 | 11.4579(4) | 275 | 11.4584(4) | 79.2(4) | 15.1(2) | 5.7(1) |
|  | 175 | 11.4952(4) | 210 | 11.4706(4) | 245 | 11.4578(4) | 280 | 11.4586(4) |  |  |  |
|  | 180 | 11.4946(4) | 215 | 11.4618(4) | 250 | 11.4577(4) | 285 | 11.4590(4) |  |  |  |
|  | 185 | 11.4939(4) | 220 | 11.4595(4) | 255 | 11.4578(4) | 290 | 11.4594(4) |  |  |  |
|  | 190 | 11.4928(4) | 225 | 11.4585(4) | 260 | 11.4578(4) | 295 | 11.4597(4) |  |  |  |
|  | 195 | 11.4899(4) | 230 | 11.4581(4) | 265 | 11.4580(4) | 300 | 11.4602(4) |  |  |  |
|  | 200 | 11.4845(4) | 235 | 11.4580(4) | 270 | 11.4581(4) |  |  |  |  |  |


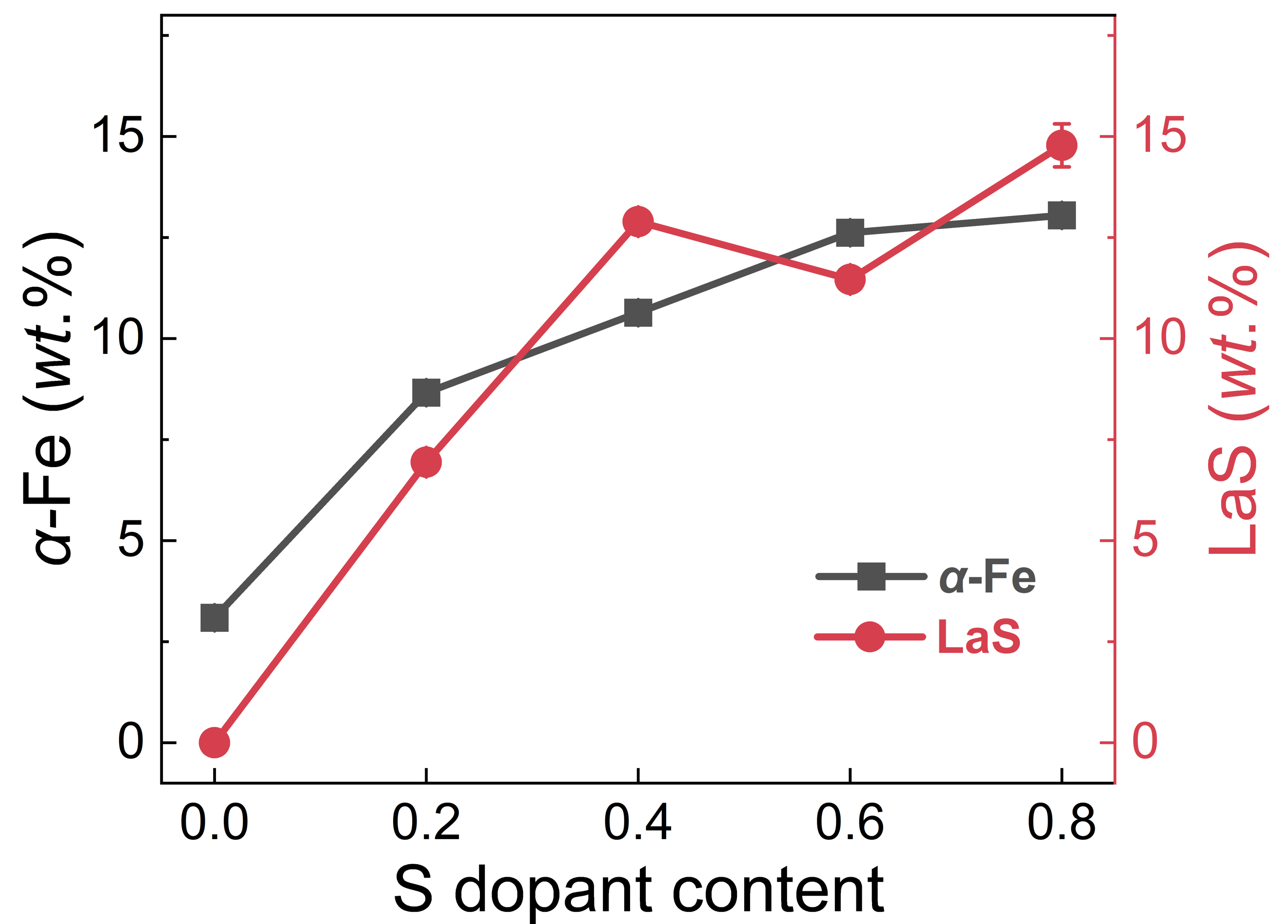


**Figure S9.** Fraction of *α*-Fe and LaS based impurity phases for different S dopant.


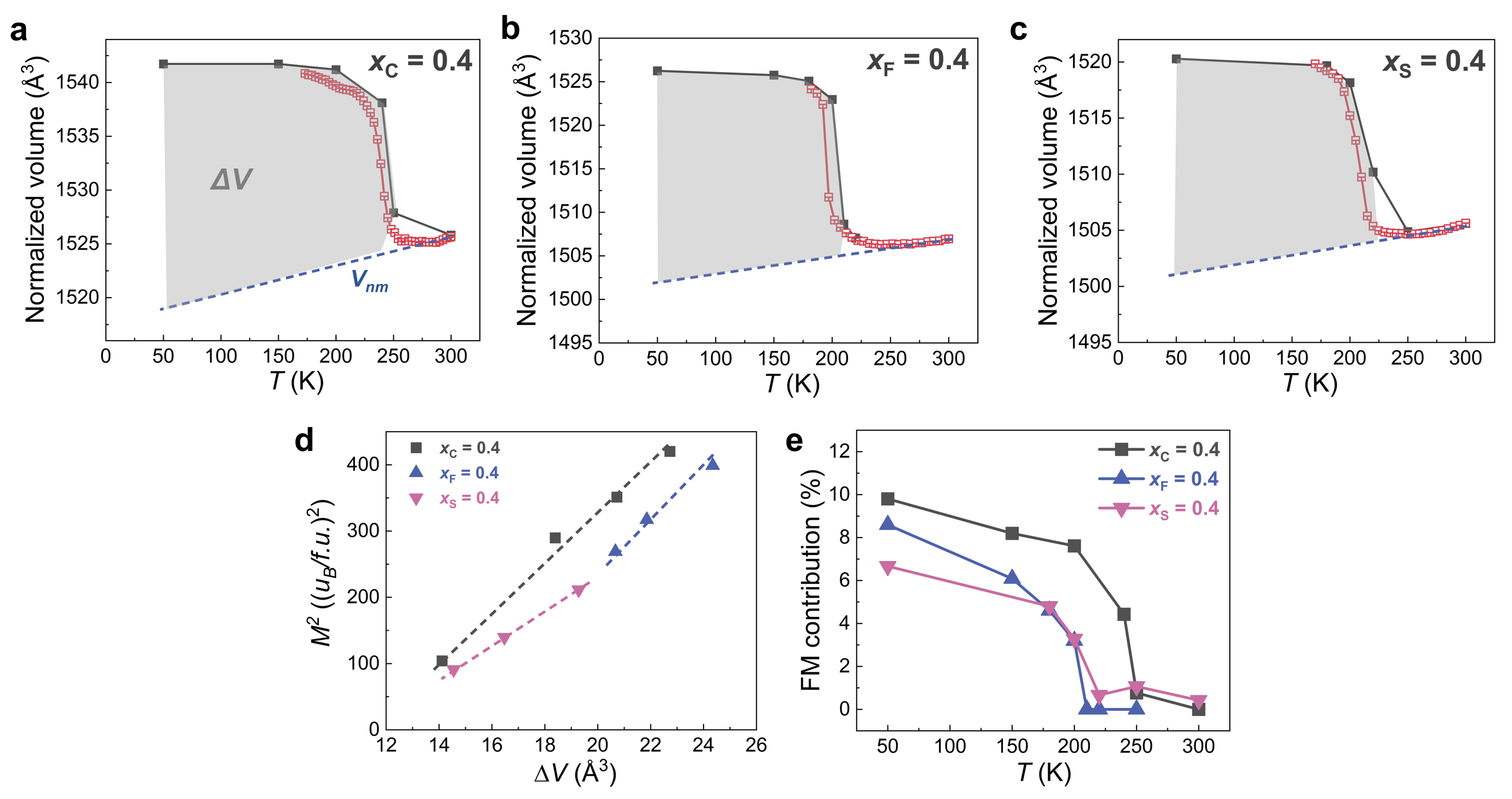


**Figure S10.** Experimental and calculated volume of unit cell in different temperature for (a) LaFe_11.6_Si_1.4_C_0.4_, (b) LaFe_11.6_Si_1.4_F_0.4_, (c) LaFe_11.6_Si_1.4_S_0.4_ samples. The calculated non-magnetic unit cell volume (*V_nm_*) is based on the Debye-Grüneisen model which characterizes the phonon contribution and consequently the volumetric order parameter Δ*V*. Note that the *V_nm_* is extrapolated by ignoring the low temperature levelling. The difference between experimental *V* and *V_nm_* reflects the magnetic contribution. (d) Square of total magnetic moment per unit cell *M*^2^ as a function of Δ*V* for different samples. (e) FM contribution percentage in different modified samples, determined from temperature-dependent ND patterns in specific diffraction peaks ((531) plane for *x*_C_ = 0.4 and *x*_F_ = 0.4; (422) plane for *x*_S_ = 0.4).


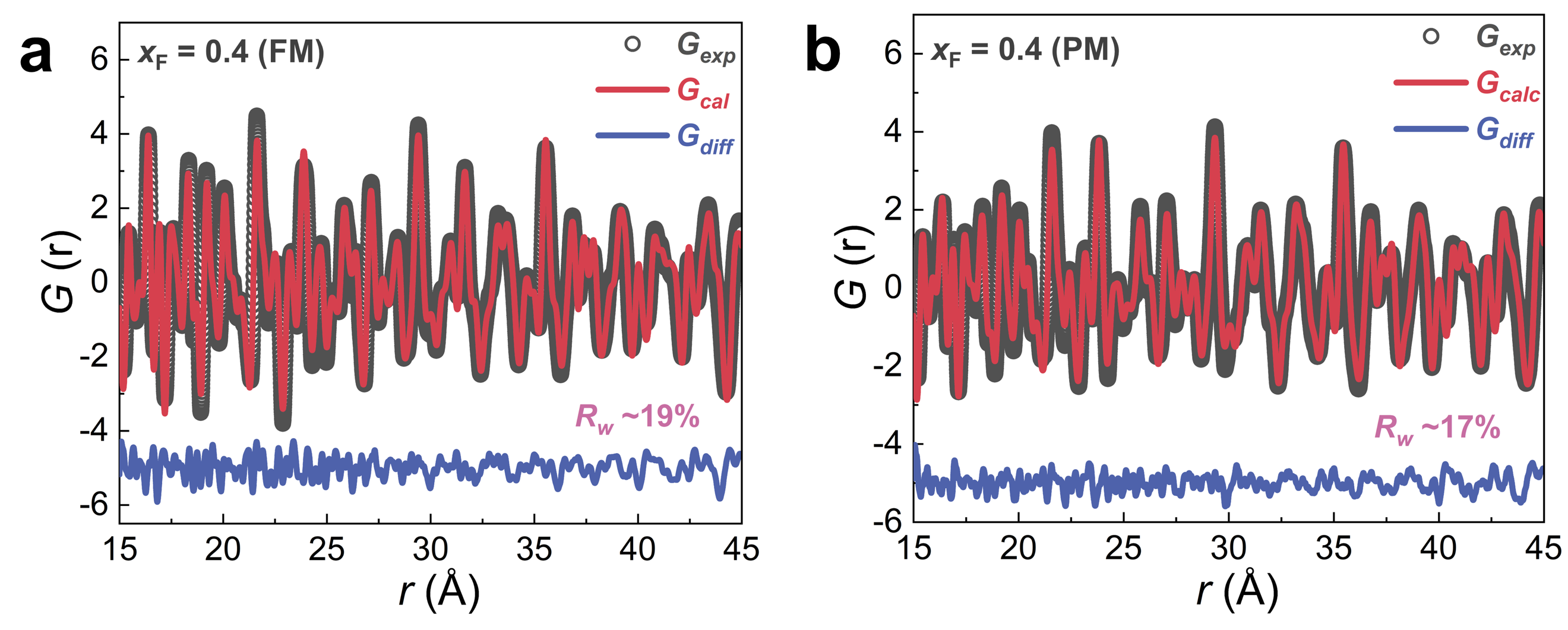


**Figure S11.** Experimental (open circle) and fitted (lines) PDF patterns for (a) FM state at 150 K and (b) PM state at 250 K in long *r* ranges for the LaFe_11.6_Si_1.4_F_0.4_ sample. The difference curves between the observed and calculated PDF patterns are shown at the bottom.


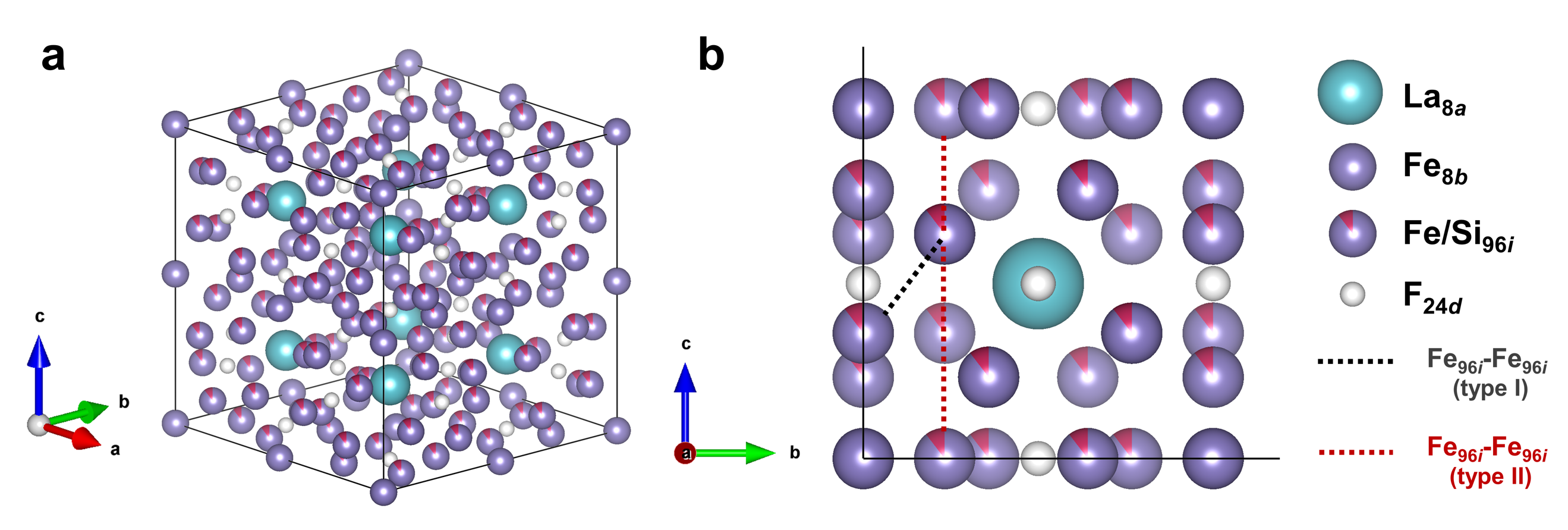


**Figure S12.** (a) Schematic diagram of the atomic structure in one unit cell for the F-doped La(Fe,Si)_13_ based compound. (b) Projection view from a direction in 1/8 part of one unit cell for the F-doped La(Fe,Si)_13_ based compound. Atoms in different positions such as 8*a*, 8*b*, 96*i* and 24*d* have been marked in different colors. And two types of atomic distances (type I and type II) of Fe_96_*_i_*-Fe_96_*_i_* have also been marked by dashed lines.
